# Supplementary material for: Noradrenaline tracks emotional modulation of attention in human amygdala
Source: Curr Biol. Author manuscript; Available in PMC 2024 Apr 9. (PMC10957395; doi:10.1016/j.cub.2023.09.074)
Supplement: Supplementary Material [file NIHMS1967216-supplement-Supplementary_Material.pdf]

Current Biology

Report

Noradrenaline tracks emotional modulation of attention in human amygdala

Highlights

- Sub-second neuromodulator dynamics can be measured using clinical depth electrodes
- Noradrenaline dynamics in human amygdala reflect attention and arousal
- Coupling between noradrenaline and pupil dilation depends on cognitive state

Authors

Dan Bang, Yi Luo, Leonardo S. Barbosa, ..., Kenneth T. Kishida, Mark R. Witcher, P. Read Montague

Correspondence

danbang@cfin.au.dk (D.B.), read@vtc.vt.edu (P.R.M.)

In brief

Bang et al. demonstrate that electrochemical estimates of sub-second neuromodulator dynamics can be obtained in humans using clinical depth electrodes. They show that noradrenaline tracks the emotional modulation of attention and that the coupling between noradrenaline and pupil dilation—a common behavioral proxy for noradrenaline—depends on cognitive state.

Bang et al., 2023, Current Biology *33*, 5003–5010
November 20, 2023 © 2023 The Author(s). Published by Elsevier Inc.
https://doi.org/10.1016/j.cub.2023.09.074

CellPress

Current Biology

CellPress
OPEN ACCESS

Report

Noradrenaline tracks emotional modulation of attention in human amygdala

Dan Bang,[1,2,3,4,15,16,*] Yi Luo,[4,5,15] Leonardo S. Barbosa,[4,6,15] Seth R. Batten,[4,15] Beniamino Hadj-Amar,[7,15] Thomas Twomey,[4] Natalie Melville,[4] Jason P. White,[4] Alexis Torres,[8] Xavier Celaya,[8] Priya Ramaiah,[9] Samuel M. McClure,[8] Gene A. Brewer,[8] Robert W. Bina,[9] Terry Lohrenz,[4] Brooks Casas,[4,10] Pearl H. Chiu,[4,10] Marina Vannucci,[7] Kenneth T. Kishida,[11,12] Mark R. Witcher,[4,13] and P. Read Montague[2,4,14,*]

[1]Center of Functionally Integrative Neuroscience, Aarhus University, 8000 Aarhus, Denmark
[2]Wellcome Centre for Human Neuroimaging, University College London, London WC1N 3BG, UK
[3]Department of Experimental Psychology, University of Oxford, Oxford OX2 6GG, UK
[4]Fralin Biomedical Research Institute at VTC, Virginia Tech, Roanoke, VA 24016, USA
[5]Shanghai Key Laboratory of Mental Health and Psychological Crisis Intervention, East China Normal University, Shanghai 200050, China
[6]Department of Psychiatry, University of Wisconsin-Madison, Madison, WI 53719, USA
[7]Department of Statistics, Rice University, Houston, TX 77005, USA
[8]Department of Psychology, Arizona State University, Tempe, AZ 85281, USA
[9]Department of Neurosurgery, Banner University Medical Center, Phoenix, AZ 85006, USA
[10]Department of Psychology, Virginia Tech, Blacksburg, VA 24060, USA
[11]Department of Physiology and Pharmacology, Wake Forest School of Medicine, Winston-Salem, NC 27101, USA
[12]Department of Neurosurgery, Wake Forest School of Medicine, Winston-Salem, NC 27101, USA
[13]Division of Neurosurgery, Virginia Tech Carilion School of Medicine, Roanoke, VA 24014, USA
[14]Department of Physics, Virginia Tech, Blacksburg, VA 24061, USA
[15]These authors contributed equally
[16]Lead contact
*Correspondence: danbang@cfin.au.dk (D.B.), read@vtc.vt.edu (P.R.M.)
https://doi.org/10.1016/j.cub.2023.09.074

SUMMARY

The noradrenaline (NA) system is one of the brain's major neuromodulatory systems; it originates in a small midbrain nucleus, the locus coeruleus (LC), and projects widely throughout the brain.[1,2] The LC-NA system is believed to regulate arousal and attention[3,4] and is a pharmacological target in multiple clinical conditions.[5–7] Yet our understanding of its role in health and disease has been impeded by a lack of direct recordings in humans. Here, we address this problem by showing that electrochemical estimates of sub-second NA dynamics can be obtained using clinical depth electrodes implanted for epilepsy monitoring. We made these recordings in the amygdala, an evolutionarily ancient structure that supports emotional processing[8,9] and receives dense LC-NA projections,[10] while patients (n = 3) performed a visual affective oddball task. The task was designed to induce different cognitive states, with the oddball stimuli involving emotionally evocative images,[11] which varied in terms of arousal (low versus high) and valence (negative versus positive). Consistent with theory, the NA estimates tracked the emotional modulation of attention, with a stronger oddball response in a high-arousal state. Parallel estimates of pupil dilation, a common behavioral proxy for LC-NA activity,[12] supported a hypothesis that pupil-NA coupling changes with cognitive state,[13,14] with the pupil and NA estimates being positively correlated for oddball stimuli in a high-arousal but not a low-arousal state. Our study provides proof of concept that neuromodulator monitoring is now possible using depth electrodes in standard clinical use.

RESULTS

Electrochemistry on clinical depth electrodes

Recent years have seen the novel application of electrochemistry during awake deep brain stimulation (DBS) surgery, in which electrodes are implanted for the management of movement disorder symptoms (e.g., Parkinson's disease).[15–19] While providing the first estimates of fast neuromodulator release from the human brain, these recordings require custom-made carbon-fiber electrodes and have naturally been constrained to the basal ganglia, mainly dorsal striatum, by the surgical procedure. However, an accurate understanding of neuromodulatory systems in human health and disease requires an ability to study their function across the brain,[20,21] especially in the case of the locus coeruleus (LC)-noradrenaline (NA) system, which plays a minimal role in the regulation of striatal activity.[2]

Here, we present an approach for obtaining sub-second neuromodulator estimates from clinical depth electrodes that are implanted throughout the brain for phase II epilepsy monitoring.[22]

© 2023 The Author(s). Published by Elsevier Inc.
This is an open access article under the CC BY license (http://creativecommons.org/licenses/by/4.0/).

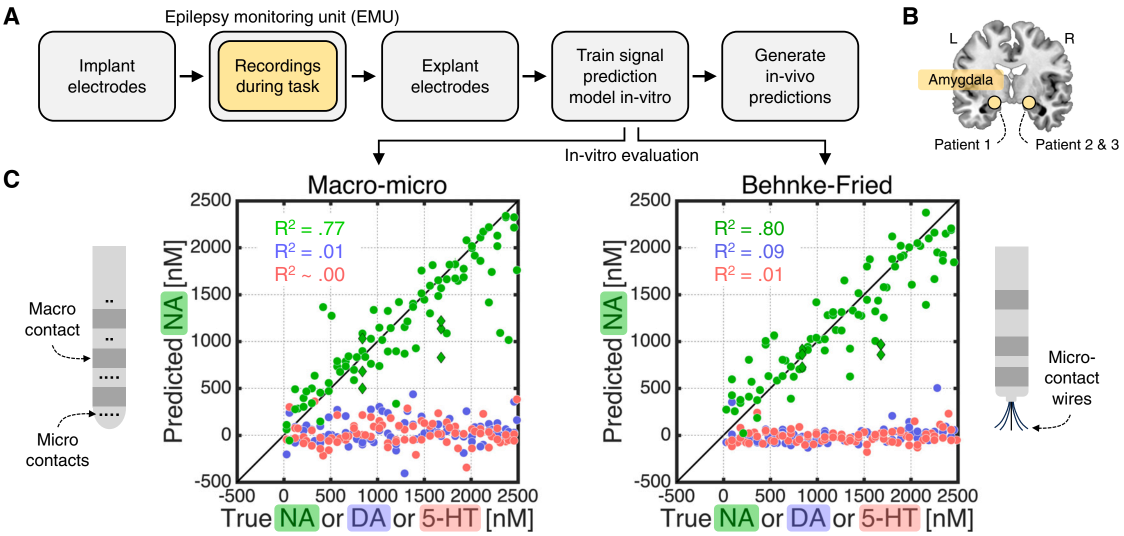

Figure 1. Electrochemistry on clinical depth electrodes

(A) Sketch of electrochemical approach.

(B) We made electrochemical recordings in the amygdala of three patients while they performed the task shown in Figure 2A.

(C) *In vitro* evaluation of electrochemical approach. The macro-micro electrodes were explanted from the three patients who performed the task in Figure 2A. The Behnke-Fried electrodes were explanted from the amygdala of three patients at another hospital and were included to demonstrate generalizability. Dots indicate the average predicted NA concentration (nanomoles, nM) for single-analyte solutions that only contained NA (green), DA (blue), or 5-HT (red). Green diamonds indicate mixture solutions that contained DA and/or 5-HT in addition to NA. Predictions were from a 10-fold cross-validation and pooled across patients. R^2 values were obtained by regressing the predicted NA concentration against the true NA, DA, or 5-HT concentration. Error bars represent 95% confidence intervals but are not visible at this scale. See Figure S1 for *in vitro* evaluations for DA and 5-HT.

Patients with medication-resistant epilepsy can become eligible for ablation of epileptic foci. To help localize such foci, depth electrodes are implanted in brain regions relevant to each patient's condition and neuronal activity is tracked over multiple days in an epilepsy monitoring unit (EMU). For years neuroscientists have utilized these electrodes for intracranial electrophysiology[23]—we show that they can be used for intracranial electrochemistry, too.

The workflow is summarized in Figure 1A. In brief, the neurosurgeon implants depth electrodes (here, Ad-Tech macro-micro) into potential epileptic loci. During their stay in the EMU, the patient performs a research task while an electrode in a region of interest is used for voltammetric recordings. Once epilepsy monitoring is complete, the neurosurgeon explants the electrodes. A signal prediction model is then created by exposing the relevant electrode to labeled concentrations of NA, dopamine (DA), serotonin (5-HT), and pH in a controlled *in vitro* setting. Finally, to generate *in vivo* neuromodulator estimates, the signal prediction model is applied to the voltammetric recordings from the patient brain.

We applied this approach in three patients using electrodes implanted in the amygdala (Figure 1B), an evolutionarily conserved structure that supports emotional processing[8,9] and receives dense LC-NA projections.[10] *In vitro* evaluation using out-of-training data showed that our approach can accurately detect NA and does not confuse NA with DA or 5-HT (Figure 1C, left). Our approach also generalized to another type of depth electrode explanted from the amygdala of three patients at another hospital (Ad-Tech Behnke-Fried; Figure 1C, right).

Visual affective oddball task

The three patients performed a visual affective oddball task (Figure 2A). The task involved the rapid presentation of unique images from the International Affective Picture System (IAPS)[11] as surprising (oddball) stimuli and a checkerboard image as the standard stimulus. The IAPS images were selected based on the content ratings provided by the IAPS and grouped within a block design such that every block contained images of the same emotional category. There were four emotionally evocative blocks, which varied in terms of valence (negative versus positive) and arousal (low versus high), and two emotionally neutral blocks. To keep patients engaged, they were told to press a button whenever an oddball stimulus was shown.

In parallel to the electrochemical recordings, we monitored pupil dilation, a widely used behavioral proxy for activity in the LC-NA system.[12] The EMU setting meant that we did not have full control over extraneous variables, such as head position, eye movement, and room lighting, which can affect pupil dilation. We therefore also ran the task in a group of control subjects (n = 17) in a standard lab setting and used correspondence between the patient and the control data as an indicator of data quality.

Behavioral validation of experimental framework

To validate the pupil data from the EMU and the task, we first tested for an oddball pupil dilation response (PDR), by applying a linear mixed-effects regression in which we predicted the pupil estimate at each time point using stimulus type (standard = −1; oddball = 1). This analysis returned an oddball PDR: pupil dilation was larger for oddball than standard stimuli, and this difference was largest around 1.2 s after stimulus onset (Figure 2B, top).

CellPress
OPEN ACCESS

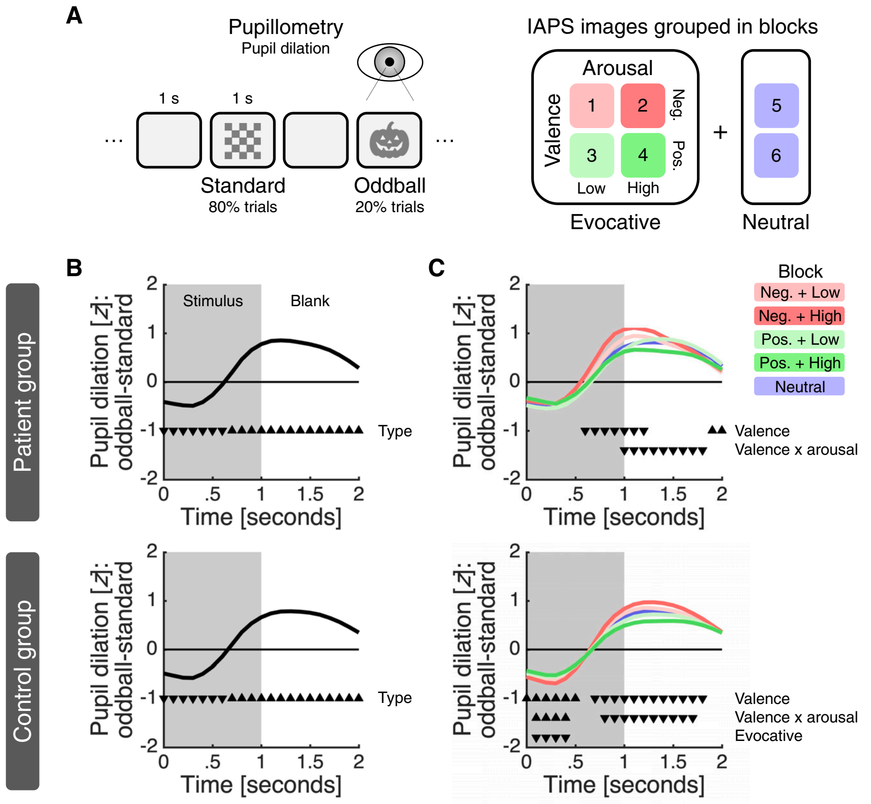

Figure 2. Experimental framework and behavioral results

(A) Patients performed a visual affective oddball task while we simultaneously measured NA in the amygdala and pupil dilation with sub-second temporal resolution. The task was divided into six blocks of 100 images; the images were shown for 1 s and separated by 1 s blank intervals. Within each block, 80% of the images were a checkerboard image (standard) and the remaining 20% were unique IAPS images (oddball). See Figure S2A for IAPS content ratings and measured illuminance for the different image sets.

(B) Oddball PDR for (top) patients and (bottom) controls across all trials.

(C) Oddball PDR for (top) patients and (bottom) controls within each condition.

(B and C) For each trial, we smoothed the time series using a 0.5 s causal filter, Z scored the smoothed data and removed any linear drift. Lines indicate the difference between the average time series for oddball and standard trials. Triangles indicate the significance ($p < 0.050$) of a predictor and their direction indicates the sign of the effect (down, negative; up, positive). Only significant predictors are shown. See Figure S2B for an analysis in raw units, which also considers image illuminance and trial history.

We next analyzed oddball trials only, now predicting the pupil estimates using emotional valence (negative = −1; neutral = 0; positive = 1), emotional arousal (low = −1; neutral = 0; high = 1), emotionally evocative (neutral = −1; evocative = 1), and an interaction between valence and arousal. The valence, arousal, and evocative terms were specified at the block level as per our task design (Figure 2A); valence and arousal were considered features of blocks involving emotionally evocative IAPS images only (Figure S2A), with the evocative term capturing any difference between these blocks and emotionally neutral blocks. This analysis identified a main effect of valence and an interaction between valence and arousal, with pupil dilation strongest for IAPS images characterized by negative valence and high arousal (Figure 2C, top). In further support of the quality of the pupil data collected in the EMU, these effects closely resembled those observed in the control group (Figures 2B and 2C, bottom). Finally, we examined button presses. Indicative of a high level of task engagement, patients detected nearly all oddball stimuli (patients, 94%–100%; controls, 96%–100%). An analysis of the impact of the task factors on reaction times did not return any effects in the patient group, but it identified an effect of arousal in the larger control group, with responses being slower in high-arousal blocks (Figure S2C).

NA estimates track the emotional modulation of attention

Our task was designed to probe attention-related processes, by randomly interspersing the repeated presentation of a checkerboard image with IAPS images, and to induce different cognitive states, by grouping the emotional content of the IAPS images within a block design (Figure 2A). To quantify the impact of these factors on NA dynamics, we employed a linear mixed-effects regression in which we predicted single-trial estimates of the NA response around stimulus presentation using stimulus type, emotional valence, emotional arousal, emotionally evocative, and interactions between these terms.

This analysis identified a positive main effect of arousal and a positive interaction between stimulus type and arousal (Figure 3A; arousal, $t(1,790) = 3.34$, $p < 0.001$; type × arousal, $t(1,790) = 4.35$, $p < 0.001$). To unpack these effects, we adopted a simple-effects approach, quantifying the impact of arousal for each stimulus type and the impact of stimulus type for each level of arousal. Indicating that NA is modulated by emotional content, we found that the estimated NA response for oddball stimuli was higher in high-arousal than low-arousal blocks (Figure 3B; effect of arousal for oddball stimuli, $t(1,790) = 4.30$, $p < 0.001$), whereas the estimated NA response for standard stimuli did not differ between these blocks (Figure 3B; effect of arousal for standard stimuli, $t(1,790) = -1.13$, $p = 0.259$). We note that the positive effect of arousal for oddball stimuli remained after controlling for reaction times in an analysis of oddball trials only (arousal, $t(346) = 4.00$, $p < 0.001$). Indicating that these effects reflect contextual modulation of the attentional salience of surprising stimuli, we found that the estimated NA response was higher for oddball than standard stimuli in high-arousal blocks but lower in low-arousal blocks (Figure 3B; effect of type for high arousal, $t(1,790) = 2.26$, $p = 0.024$; effect of type for low arousal, $t(1,790) = -2.09$, $p = 0.037$). While a stronger response for oddball than

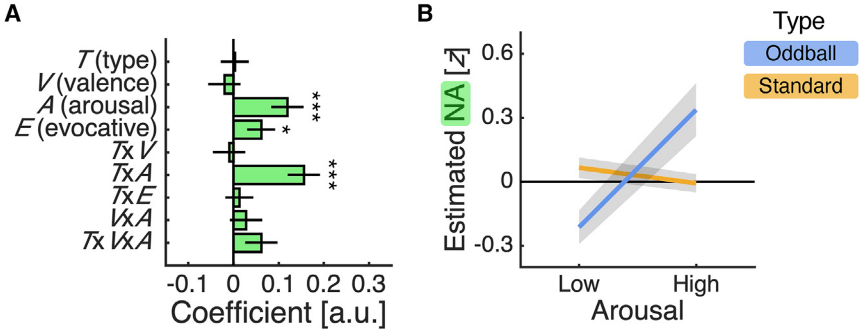

Figure 3. NA estimates track the emotional modulation of attention

(A) Regression coefficients ± SE from linear mixed-effects regression described in main text. *p < 0.050; **p < 0.010; ***p < 0.001.

(B) Single-trial NA estimates (mean ± SE) separated by emotional arousal and stimulus type.

(A and B) Single-trial NA estimates were calculated as the mean NA estimate over a 1 s window centered on stimulus onset minus the mean NA estimate over the preceding 0.5 s and Z scored across trials for each patient. See Figure S3 for analysis of DA and 5-HT estimates.

standard stimuli is in line with theories of LC-NA function, there is, to our knowledge, no account that predicts the opposite relationship. In addition to these arousal-related effects, the analysis identified a positive main effect of emotionally evocative (Figure 3A; evocative, $t(1,790) = 1.98$, $p = 0.048$), indicating that the estimated NA response was overall higher in emotionally evocative than emotionally neutral blocks.

While not the focus of our study, our electrochemical approach also returned estimates of DA and 5-HT. Applying the same analysis as for NA, we found that the estimated DA response for oddball stimuli was higher in emotionally evocative than emotionally neutral blocks, whereas the estimated 5-HT response was overall lower in emotionally evocative than emotionally neutral blocks (Figure S3). The DA and 5-HT results help clarify the specificity of the NA results, with the estimated NA response reflecting contextual modulation of attention in the most emotion-specific manner.

Coupling between pupil and NA estimates varies with emotional arousal

We next turned to the relationship between the pupil and NA estimates. The presence of pupil-NA coupling would both support the use of pupillometry as an indirect measure of LC-NA activity[12] and corroborate our electrochemical approach. In addition to providing the first-ever characterization of the link between pupil dilation and NA in a key LC-NA projection target, these data may also help us understand how pupil-NA coupling changes with cognitive state.[13,14] We used the estimated pupil and NA time series across all trials to test for a general presence of pupil-NA coupling and—guided by the NA results—the time series for oddball trials in low- and high-arousal blocks to test for a dependence of pupil-NA coupling on cognitive state. For each condition of interest, we quantified the relationship between the estimated pupil and NA time series averaged across patients in two ways. First, we conducted simple (Pearson) correlations. Second, as simple correlations cannot capture time-varying relationships, we also fitted a hidden Markov model (HMM), a popular approach for unsupervised statistical learning of multivariate time series data that can identify changes in correlation without making a priori assumptions about when these changes might occur.[24,25]

In support of pupillometry as an indirect measure of LC-NA activity and our electrochemical approach, we found a positive correlation between the estimated pupil and NA time series averaged across all trials (Figure 4, top left; $r = 0.37$, $p = 0.004$). In addition, in line with the hypothesis that pupil-NA coupling depends on cognitive state, we found a negative correlation between the average estimated pupil and NA time series for low-arousal oddball trials (Figure 4, top middle; $r = -0.59$, $p < 0.001$) but a positive correlation for high-arousal oddball trials (Figure 4, top right; $r = 0.74$, $p < 0.001$). Importantly, the HMM approach showed that the positive correlation across all trials was strongest (Figure 4, bottom left), and that the difference in the sign of the correlation between low-arousal and high-arousal oddball trials was largest (Figure 4, bottom middle versus bottom right), around stimulus presentation.

DISCUSSION

The LC-NA system affects neural activity across the brain.[1,2] This influence, often summarized as an impact on global brain states,[26,27] has been the target of theoretical models cast at different levels of description, including neuronal processes such as gain control and cognitive processes such as arousal and attention.[3,4,28,29] Clinically, the LC-NA system is implicated in multiple conditions, perhaps most prominently attention-deficit/hyperactivity disorder.[7,30–32] Yet there has not been a way to directly measure NA in humans. Here, we provide proof of concept that electrochemical estimates of sub-second NA dynamics can be obtained using clinical depth electrodes implanted for epilepsy monitoring.

By applying our electrochemical approach in human amygdala together with a visual affective oddball task and pupillometry, we tested theoretical predictions about the role of NA in arousal and attention and examined the relationship between pupil dilation and NA. In our task, IAPS images of a particular emotional category were shown on 20% of trials (oddball) and a checkerboard image on the remaining 80% of trials (standard). In line with a hypothesis that NA tracks the emotional modulation of attention, we found that the NA response around stimulus presentation discriminated (1) between IAPS images that induced states of high versus low emotional arousal and (2) between the surprising IAPS images and the non-surprising checkerboard image within these two emotional states.

Comparison of the pupil and NA estimates supported the use of pupil dilation as a window into the LC-NA system.[12] In line with earlier reports that activity in LC neurons[33] and LC-NA axonal projections[34] can predict pupil dilation, we found a positive pupil-NA correlation for all trials regardless of stimulus type or emotional context. However, pupil dilation is influenced by

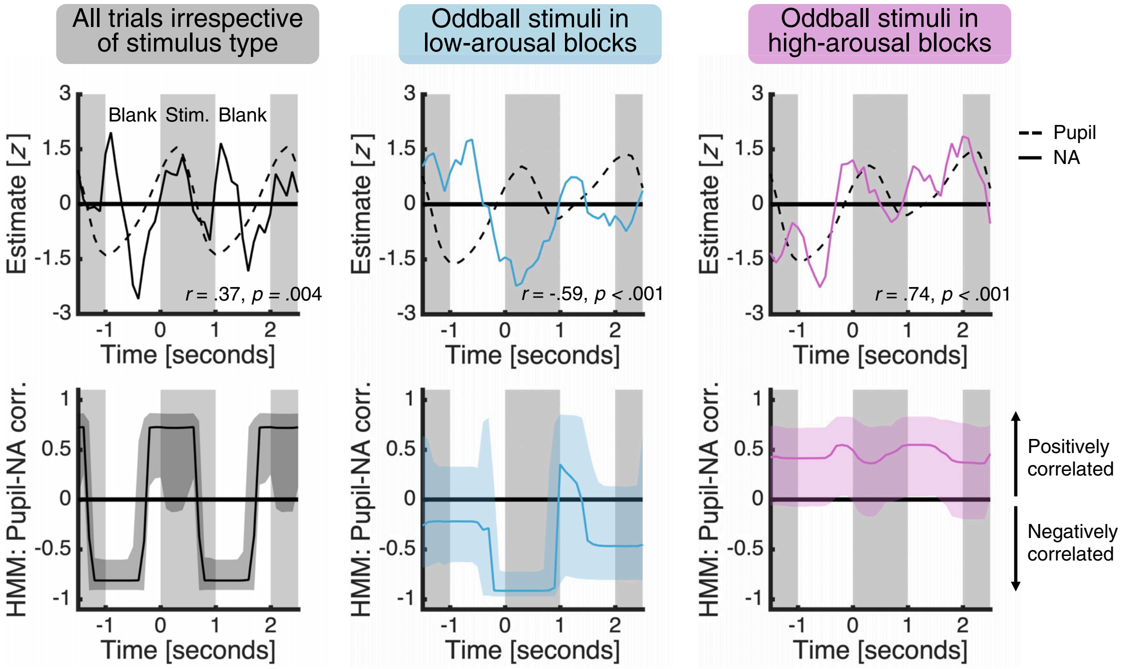

Figure 4. Coupling between pupil and NA estimates varies with emotional arousal
The panels show (top) the average estimated pupil and NA time series with simple correlation statistics reported and (bottom) the HMM estimate of pupil-NA coupling for (left) all trials irrespective of stimulus type, (middle) oddball stimuli in low-arousal blocks, and (right) oddball stimuli in high-arousal blocks. We first smoothed the time series using a 0.5 s causal filter and Z scored the smoothed data for each trial. We then averaged the time series for each condition, removed any linear drift, and scaled the average time series to have unit variance, by dividing each time point by the average of the condition-specific standard deviations over the average time series. The last step helps ensure that estimated correlations, which are sensitive to the variance of the data, are comparable across conditions. Shaded area for the HMM estimate of pupil-NA coupling indicates 95% credible interval. See Figure S4 for simple correlations for oddball stimuli in each block.

multiple systems,[12] and there is evidence that pupil-NA coupling may change with cognitive state.[13,14] Indeed, we found a positive pupil-NA correlation for the surprising IAPS images in a high-arousal state but a negative correlation in a low-arousal state. While a negative correlation is unexpected, it can arise when two systems, such as the pupil and LC-NA systems,[35] are only driven by partially overlapping inputs. If there is not a strong common input to synchronize the systems, then the non-overlapping inputs may drive their responses, and if these inputs happen to go in the opposite directions, then negative correlations may arise.

Prior to this study, human electrochemistry has been performed during awake DBS surgery.[15–19] With minimal deviations from the standard of care, and no reported changes in infection rates,[36] a custom-made carbon-fiber electrode can be inserted along the same path as the clinical electrodes used for functional mapping of the DBS target. The capacity to perform human electrochemistry in the EMU complements the DBS-based approach in multiple ways. It enables electrochemical recordings from a wider range of neural structures, and it allows for the study of physiological, behavioral, and cognitive processes that cannot be probed within the constraints of an acute surgical setting. For example, the LC-NA system is a pharmacological target in sleep disorders,[37] and it will be possible to monitor NA as patients transition between wakefulness and sleep. Similarly, a proportion of patients will be undergoing pharmacotherapy, such as selective serotonin reuptake inhibitors (SSRIs) or serotonin-NA reuptake inhibitors (SNRIs), for psychiatric symptoms, and it will be possible to compare neuromodulation before and after drug intake.

The brain's major neuromodulatory systems support critical physiological, behavioral, and cognitive processes and have been implicated in a variety of clinical conditions. While high-precision methods for studying neuromodulation are available in animals,[38,39] similar advances are needed in humans to accelerate translation and advance our understanding of health and disease. The neuromodulatory machinery may be conserved across species, but it also operates on species-unique neural hardware and cognitive software in humans. By showing that neuromodulator estimates can be obtained from depth electrodes already in standard clinical use in the conscious human brain, our study opens the door to a new area of research on the neuromodulatory basis of human health and disease.

STAR★METHODS

Detailed methods are provided in the online version of this paper and include the following:

- KEY RESOURCES TABLE
- RESOURCE AVAILABILITY
  - Lead contact
  - Materials availability
  - Data and code availability

- EXPERIMENTAL MODEL AND STUDY PARTICIPANT DETAILS
  - Patients
  - Controls
- METHOD DETAILS
  - Experimental task
  - Pupillometry
  - General description of electrochemical approach
  - Implementation of electrochemistry in current study
- QUANTIFICATION AND STATISTICAL ANALYSIS
  - Standardization
  - Mixed-effects regression
  - Hidden Markov model

SUPPLEMENTAL INFORMATION

Supplemental information can be found online at https://doi.org/10.1016/j.cub.2023.09.074.

ACKNOWLEDGMENTS

We thank the patient volunteers and the research and surgical EMU and nursing staff at the Carilion Clinic and the Banner University Medical Center for invaluable support and cooperation. This work was supported by the Lundbeck Foundation (D.B., R368-2021-325), the Wellcome Trust (D.B., 213630/Z/18/Z; P.R.M., 091188/Z/10/Z), the Swartz Foundation (P.R.M., 2019-11), NIH-NCATS (K.T.K., KL2TR001421), NIH-NIDA (K.T.K., R01-DA048096), NIH-NINDS (K.T.K. and P.R.M., R01-NS092701), NIH-NIMH (K.T.K., R01-MH121099; K.T.K., M.V., and P.R.M., R01-MH124115; P.H.C. and P.R.M., R01-MH122512; B.C. and P.R.M., R01-MH122948; P.H.C., R01-127773), VA-RR&D (B.C., D2354R), a donation by Steve Neumann (A.T., X.C., and G.A.B.), and a Virginia Tech Foundation Seale Innovation Award (P.R.M., FY22). The Wellcome Centre for Human Neuroimaging is supported by core funding from the Wellcome Trust (203147/Z/16/Z). For the purpose of open access, the authors have applied a CC BY public copyright license to any Author Accepted Manuscript version arising from this submission.

AUTHOR CONTRIBUTIONS

Conceptualization, D.B., Y.L., T.L., B.C., P.H.C., K.T.K., M.R.W., and P.R.M.; data curation, D.B., Y.L., L.S.B., S.R.B., T.T., N.M., J.P.W., A.T., X.C., and P.R.; formal analysis, D.B., L.S.B., B.H.-A., T.L., M.V., and P.R.M.; funding acquisition, D.B., B.C., P.H.C., M.V., K.T.K., and P.R.M.; investigation, D.B., Y.L., S.R.B., N.M., J.P.W., A.T., X.C., R.W.B., and M.R.W.; methodology, D.B., L.S.B., B.H.-A., T.T., J.P.W., S.M.M., G.A.B., R.W.B., T.L., M.V., M.R.W., and P.R.M.; project administration, S.R.B., N.M., and P.R.; resources, R.W.B., M.R.W., and P.R.M.; software, D.B., L.S.B., B.H.-A., T.T., J.P.W., T.L., M.V., and P.R.M.; supervision, M.V. and P.R.M.; validation, D.B., L.S.B., T.T., J.P.W., T.L., and P.R.M.; visualization, D.B.; writing – original draft, D.B. and P.R.M.; writing – review & editing, D.B., Y.L., L.S.B., S.R.B., B.H.-A., T.T., N.M., J.P.W., G.A.B., R.W.B., T.L., M.V., K.T.K., M.R.W., and P.R.M.

DECLARATION OF INTERESTS

The authors declare no competing interests.

INCLUSION AND DIVERSITY

We support inclusive, diverse, and equitable conduct of research.

Received: December 14, 2022
Revised: September 1, 2023
Accepted: September 29, 2023
Published: October 23, 2023

REFERENCES

1. Poe, G.R., Foote, S., Eschenko, O., Johansen, J.P., Bouret, S., Aston-Jones, G., Harley, C.W., Manahan-Vaughan, D., Weinshenker, D., Valentino, R., et al. (2020). Locus coeruleus: a new look at the blue spot. Nat. Rev. Neurosci. *21*, 644–659. https://doi.org/10.1038/s41583-020-0360-9.
2. Schwarz, L.A., and Luo, L. (2015). Organization of the locus coeruleus-norepinephrine system. Curr. Biol. *25*, R1051–R1056. https://doi.org/10.1016/j.cub.2015.09.039.
3. Sara, S.J., and Bouret, S. (2012). Orienting and reorienting: the locus coeruleus mediates cognition through arousal. Neuron *76*, 130–141. https://doi.org/10.1016/j.neuron.2012.09.011.
4. Aston-Jones, G., and Cohen, J.D. (2005). An integrative theory of locus coeruleus-norepinephrine function: adaptive gain and optimal performance. Annu. Rev. Neurosci. *28*, 403–450. https://doi.org/10.1146/annurev.neuro.28.061604.135709.
5. Otte, C., Gold, S.M., Penninx, B.W., Pariante, C.M., Etkin, A., Fava, M., Mohr, D.C., and Schatzberg, A.F. (2016). Major depressive disorder. Nat. Rev. Dis. Prim. *2*, 1–21. https://doi.org/10.1038/nrdp.2016.65.
6. Craske, M.G., Stein, M.B., Eley, T.C., Milad, M.R., Holmes, A., Rapee, R.M., and Wittchen, H.-U. (2017). Anxiety disorders. Nat. Rev. Dis. Primers *3*, 17024, https://doi.org/10.1038/nrdp.2017.24.
7. del Campo, N., Chamberlain, S.R., Sahakian, B.J., and Robbins, T.W. (2011). The roles of dopamine and noradrenaline in the pathophysiology and treatment of attention-deficit/hyperactivity disorder. Biol. Psychiatry *69*, e145–e157. https://doi.org/10.1016/j.biopsych.2011.02.036.
8. Phelps, E.A., and LeDoux, J.E. (2005). Contributions of the amygdala to emotion processing: from animal models to human behavior. Neuron *48*, 175–187. https://doi.org/10.1016/j.neuron.2005.09.025.
9. Phelps, E.A. (2006). Emotion and cognition: insights from studies of the human amygdala. Annu. Rev. Psychol. *57*, 27–53. https://doi.org/10.1146/annurev.psych.56.091103.070234.
10. McCall, J.G., Siuda, E.R., Bhatti, D.L., Lawson, L.A., McElligott, Z.A., Stuber, G.D., and Bruchas, M.R. (2017). Locus coeruleus to basolateral amygdala noradrenergic projections promote anxiety-like behavior. eLife *6*, e18247, https://doi.org/10.7554/eLife.18247.
11. Lang, P.J., Bradley, M.M., and B.N.. (2008). Cuthbert International Affective Picture System (IAPS): Affective Ratings of Pictures and Instruction Manual (University of Florida).
12. Joshi, S., and Gold, J.I. (2019). Pupil size as a window on neural substrates of cognition. Trends Cogn. Sci. *24*, 466–480. https://doi.org/10.31234/osf.io/dvsme.
13. Megemont, M., McBurney-Lin, J., and Yang, H. (2022). Pupil diameter is not an accurate real-time readout of locus coeruleus activity. eLife *11*, e70510, https://doi.org/10.7554/eLife.70510.
14. Yang, H., Bari, B.A., Cohen, J.Y., and O'Connor, D.H. (2021). Locus coeruleus spiking differently correlates with S1 cortex activity and pupil diameter in a tactile detection task. eLife *10*, e64327, https://doi.org/10.7554/eLife.64327.
15. Kishida, K.T., Sandberg, S.G., Lohrenz, T., Comair, Y.G., Sáez, I., Phillips, P.E.M., and Montague, P.R. (2011). Sub-second dopamine detection in human striatum. PLoS One *6*, 4–8. https://doi.org/10.1371/journal.pone.0023291.
16. Kishida, K.T., Saez, I., Lohrenz, T., Witcher, M.R., Laxton, A.W., Tatter, S.B., White, J.P., Ellis, T.L., Phillips, P.E.M., and Montague, P.R. (2016). Subsecond dopamine fluctuations in human striatum encode superposed error signals about actual and counterfactual reward. Proc. Natl. Acad. Sci. USA *113*, 200–205. https://doi.org/10.1073/pnas.1513619112.
17. Moran, R.J., Kishida, K.T., Lohrenz, T., Saez, I., Laxton, A.W., Witcher, M.R., Tatter, S.B., Ellis, T.L., Phillips, P.E., Dayan, P., et al. (2018). The protective action encoding of serotonin transients in the human brain. Neuropsychopharmacology *43*, 1425–1435. https://doi.org/10.1038/npp.2017.304.

18. Bang, D., Kishida, K.T., Lohrenz, T., White, J.P., Laxton, A.W., Tatter, S.B., Fleming, S.M., and Montague, P.R. (2020). Sub-second dopamine and serotonin signaling in human striatum during perceptual decision-making. Neuron *108*, 999–1010.e6. https://doi.org/10.1016/j.neuron.2020.09.015.

19. Montague, P.R., and Kishida, K.T. (2018). Computational underpinnings of neuromodulation in humans. Cold Spring Harbor Symp. Quant. Biol. *83*, 71–82. https://doi.org/10.1101/sqb.2018.83.038166.

20. Ren, J., Friedmann, D., Xiong, J., Liu, C.D., Ferguson, B.R., Weerakkody, T., DeLoach, K.E., Ran, C., Pun, A., Sun, Y., et al. (2018). Anatomically defined and functionally distinct dorsal raphe serotonin sub-systems. Cell *175*, 472–487.e20. https://doi.org/10.1016/j.cell.2018.07.043.

21. Engelhard, B., Finkelstein, J., Cox, J., Fleming, W., Jang, H.J., Ornelas, S., Koay, S.A., Thiberge, S.Y., Daw, N.D., Tank, D.W., et al. (2019). Specialized coding of sensory, motor and cognitive variables in VTA dopamine neurons. Nature *570*, 509–513. https://doi.org/10.1038/s41586-019-1261-9.

22. Rosenow, F., and Lüders, H. (2001). Presurgical evaluation of epilepsy. Brain *124*, 1683–1700. https://doi.org/10.1093/brain/124.9.1683.

23. Parvizi, J., and Kastner, S. (2018). Promises and limitations of human intracranial electroencephalography. Nat. Neurosci. *21*, 474–483. https://doi.org/10.1038/s41593-018-0108-2.

24. Rabiner, L.R. (1989). A tutorial on hidden Markov models and selected applications in speech recognition. Proc. IEEE *77*, 257–286. https://doi.org/10.1109/5.18626.

25. Zucchini, W., MacDonald, I.L., and Langrock, R. (2016). Hidden Markov Models for Time Series: An Introduction Using R, Second Edition (Chapman and Hall/CRC).

26. Berridge, C.W., and Waterhouse, B.D. (2003). The locus coeruleus–noradrenergic system: modulation of behavioral state and state-dependent cognitive processes. Brain Res. Brain Res. Rev. *42*, 33–84. https://doi.org/10.1016/S0165-0173(03)00143-7.

27. O'Donnell, J., Zeppenfeld, D., McConnell, E., Pena, S., and Nedergaard, M. (2012). Norepinephrine: a neuromodulator that boosts the function of multiple cell types to optimize CNS performance. Neurochem. Res. *37*, 2496–2512. https://doi.org/10.1007/s11064-012-0818-x.

28. Cools, R., and Arnsten, A.F.T. (2022). Neuromodulation of prefrontal cortex cognitive function in primates: the powerful roles of monoamines and acetylcholine. Neuropsychopharmacology *47*, 309–328. https://doi.org/10.1038/s41386-021-01100-8.

29. Usher, M., Cohen, J.D., Servan-Schreiber, D., Rajkowski, J., and Aston-Jones, G. (1999). The role of locus coeruleus in the regulation of cognitive performance. Science *283*, 549–554. https://doi.org/10.1126/science.283.5401.549.

30. Kim, C.H., Hahn, M.K., Joung, Y., Anderson, S.L., Steele, A.H., Mazei-Robinson, M.S., Gizer, I., Teicher, M.H., Cohen, B.M., Robertson, D., et al. (2006). A polymorphism in the norepinephrine transporter gene alters promoter activity and is associated with attention-deficit hyperactivity disorder. Proc. Natl. Acad. Sci. USA *103*, 19164–19169. https://doi.org/10.1073/pnas.0510836103.

31. Bymaster, F.P., Katner, J.S., Nelson, D.L., Hemrick-Luecke, S.K., Threlkeld, P.G., Heiligenstein, J.H., Morin, S.M., Gehlert, D.R., and Perry, K.W. (2002). Atomoxetine increases extracellular levels of norepinephrine and dopamine in prefrontal cortex of rat: a potential mechanism for efficacy in attention deficit/hyperactivity disorder. Neuropsychopharmacology *27*, 699–711. https://doi.org/10.1016/S0893-133X(02)00346-9.

32. Prince, J. (2008). Catecholamine dysfunction in attention-deficit/hyperactivity disorder: an update. J. Clin. Psychopharmacol. *28*, S39–S45. https://doi.org/10.1097/JCP.0b013e318174f92a.

33. Joshi, S., Li, Y., Kalwani, R.M., and Gold, J.I. (2016). Relationships between pupil diameter and neuronal activity in the locus coeruleus, colliculi, and cingulate cortex. Neuron *89*, 221–234. https://doi.org/10.1016/j.neuron.2015.11.028.

34. Reimer, J., McGinley, M.J., Liu, Y., Rodenkirch, C., Wang, Q., McCormick, D.A., and Tolias, A.S. (2016). Pupil fluctuations track rapid changes in adrenergic and cholinergic activity in cortex. Nat. Commun. *7*, 13289, https://doi.org/10.1038/ncomms13289.

35. Costa, V.D., and Rudebeck, P.H. (2016). More than meets the eye: the relationship between pupil size and locus coeruleus activity. Neuron *89*, 8–10. https://doi.org/10.1016/j.neuron.2015.12.031.

36. Liebenow, B., Williams, M., Wilson, T., Haq, I.U., Siddiqui, M.S., Laxton, A.W., Tatter, S.B., and Kishida, K.T. (2022). Intracranial approach for sub-second monitoring of neurotransmitters during DBS electrode implantation does not increase infection rate. PLoS One *17*, e0271348, https://doi.org/10.1371/journal.pone.0271348.

37. Mitchell, H.A., and Weinshenker, D. (2010). Good night and good luck: norepinephrine in sleep pharmacology. Biochem. Pharmacol. *79*, 801–809. https://doi.org/10.1016/j.bcp.2009.10.004.

38. Tye, K.M., and Deisseroth, K. (2012). Optogenetic investigation of neural circuits underlying brain disease in animal models. Nat. Rev. Neurosci. *13*, 251–266. https://doi.org/10.1038/nrn3171.

39. Grienberger, C., and Konnerth, A. (2012). Imaging calcium in neurons. Neuron *73*, 862–885. https://doi.org/10.1016/j.neuron.2012.02.011.

40. Van Rossum, G., and Drake, F.L. (2009). Python 3 Reference Manual (CreateSpace).

41. R Core Team (2021). R: A Language and Environment for Statistical Computing (R Foundation for Statistical Computing).

42. Carpenter, B., Gelman, A., Hoffman, M.D., Lee, D., Goodrich, B., Betancourt, M., Brubaker, M.A., Guo, J., Li, P., and Riddell, A. (2017). Stan: a probabilistic programming language. J. Stat. Software *76*, 1–32. https://doi.org/10.18637/jss.v076.i01.

43. Abadi, M., Agarwal, A., Barham, P., Brevdo, E., Chen, Z., Citro, C., Corrado, G.S., Davis, A., Dean, J., Devin, M., et al. (2015). TensorFlow: large-scale machine learning on heterogeneous systems. https://doi.org/10.48550/arXiv.1605.08695.

44. Chollet, F. (2015). Keras. GitHub. https://github.com/fchollet/keras.

45. Mathôt, S., and Vilotijević, A. (2022). Methods in cognitive pupillometry: design, preprocessing, and statistical analysis. Behav. Res. Methods *55*, 3055–3077. https://doi.org/10.3758/s13428-022-01957-7.

46. Bucher, E.S., and Wightman, R.M. (2015). Electrochemical analysis of neurotransmitters. Annu. Rev. Anal. Chem. (Palo Alto. Calif) *8*, 239–261. https://doi.org/10.1146/annurev-anchem-071114-040426.

47. Rodeberg, N.T., Sandberg, S.G., Johnson, J.A., Phillips, P.E.M., and Wightman, R.M. (2017). Hitchhiker's guide to voltammetry: acute and chronic electrodes for in vivo fast-scan cyclic voltammetry. ACS Chem. Neurosci. *8*, 221–234. https://doi.org/10.1021/acschemneuro.6b00393.

48. Phillips, P.E.M., Stuber, G.D., Heien, M.L.A.V., Wightman, R.M., and Carelli, R.M. (2003). Subsecond dopamine release promotes cocaine seeking. Nature *422*, 614–618. https://doi.org/10.1038/nature01476.

49. Heien, M.L.A.V., Johnson, M.A., and Wightman, R.M. (2004). Resolving neurotransmitters detected by fast-scan cyclic voltammetry. Anal. Chem. *76*, 5697–5704. https://doi.org/10.1021/ac0491509.

50. Zou, H., and Hastie, T. (2005). Regularization and variable selection via the elastic net. J. R. Stat. Soc. B *67*, 301–320. https://doi.org/10.1111/j.1467-9868.2005.00503.x.

51. Clark, J.J., Sandberg, S.G., Wanat, M.J., Gan, J.O., Horne, E.A., Hart, A.S., Akers, C.A., Parker, J.G., Willuhn, I., Martinez, V., et al. (2010). Chronic microsensors for longitudinal, subsecond dopamine detection in behaving animals. Nat. Methods *7*, 126–129. https://doi.org/10.1038/nmeth.1412.

52. Ismail Fawaz, H.I., Lucas, B., Forestier, G., Pelletier, C., Schmidt, D.F., Weber, J., Webb, G.I., Idoumghar, L., Muller, P.-A., and Petitjean, F. (2020). InceptionTime: finding AlexNet for time series classification. Data Min. Knowl. Disc. *34*, 1936–1962. https://doi.org/10.1007/s10618-020-00710-y.

53. He, K., Zhang, X., Ren, S., and Sun, J. (2015). Deep residual learning for image recognition. https://doi.org/10.48550/arXiv.1512.03385.

54. Kingma, D.P., and Ba, J. (2017). Adam: a method for stochastic optimization. https://doi.org/10.48550/arXiv.1412.6980.

55. van de Schoot, R., Depaoli, S., King, R., Kramer, B., Märtens, K., Tadesse, M.G., Vannucci, M., Gelman, A., Veen, D., Willemsen, J., et al. (2021). Bayesian statistics and modelling. Nat. Rev. Methods Primers *1*, 1–26. https://doi.org/10.1038/s43586-020-00001-2.

56. Lewandowski, D., Kurowicka, D., and Joe, H. (2009). Generating random correlation matrices based on vines and extended onion method. J. Multivariate Anal. *100*, 1989–2001. https://doi.org/10.1016/j.jmva.2009.04.008.

57. Kass, R.E., and Raftery, A.E. (1995). Bayes factors. J. Am. Stat. Assoc. *90*, 773–795. https://doi.org/10.1080/01621459.1995.10476572.

58. Meng, X.-L., and Wong, W.H. (1996). Simulating ratios of normalizing constants via a simple identity: a theoretical exploration. Stat. Sinica *6*, 831–860.

59. Gronau, Q.F., Singmann, H., and Wagenmakers, E.-J. (2020). bridgesampling: an R package for estimating normalizing constants. J. Stat. Software *92*, 1–29. https://doi.org/10.18637/jss.v092.i10.

STAR★METHODS

KEY RESOURCES TABLE

REAGENT or RESOURCE	SOURCE	IDENTIFIER
Chemicals, peptides, and recombinant proteins		
Noradrenaline (NA)	Sigma-Aldrich	A7257; CAS: 51-41-2
Dopamine (DA)	Sigma-Aldrich	H8502; CAS: 62-31-7
Serotonin (5-HT)	Sigma-Aldrich	H9523; CAS: 153-98-0
Reagent for PBS: NaCl	Sigma-Aldrich	S7653-1K; CAS: 7647-14-5
Reagent for PBS: KCl	Sigma-Aldrich	P9333-1K; CAS: 447-40-7
Reagent for PBS: Na2HPO	Sigma-Aldrich	S7907-1K; CAS: 7558-79-4
Reagent for PBS: KH2PO4	Sigma-Aldrich	P5655-1K; CAS: 7778-77-0
Deposited data		
Neural, pupil, and behavioral	This paper	https://github.com/danbang/article-NA-pupil-IAPS-oddball
Software and algorithms		
pCLAMP	Molecular Devices	pCLAMP 10 Axon Instruments
MATLAB	MathWorks	MATLAB R2022a
Python	Van Rossum and Drake[40]	Python 3.9.7
R	R Core Team[41]	R 4.2.0
Stan	Carpenter et al.[42]	Stan 2.21.0
TensorFlow	Abadi et al.[43]	TensorFlow 2.6.0
Keras	Chollet[44]	Keras 2.6.0
Velocity-based blink detection algorithm with cubic-spline reconstruction	Mathôt and Vilotijević[45]	https://pydatamatrix.eu/
Code for reproducing figures	This paper	https://github.com/danbang/article-NA-pupil-IAPS-oddball
HMM tutorial	This paper	https://github.com/Beniamino92/mvHMM/tree/main/HMM-NE-pupil-IAPS-oddball
Other		
Head stage	Molecular Devices	CV-7B-EC Axon Instruments
Amplifier	Molecular Devices	Multiclamp 700B Axon Instruments
A/D converter	Molecular Devices	Digidata 1440A Axon Instruments
Force function (TTL) generator	Tektronix	AFG320
Isolation transformer	Tripp Lite	IS500HG Isolation Transformer
Macro-micro electrode	Ad-Tech	MM16A-SP05X-000
Benhke-Fried electrode	Ad-Tech	MM16A-SP05X-000 (outer depth electrode) and WB09R-SP00X-0B6 (micro-wire bundle)
Eye-tracking system	Tobii	Tobii Pro Spectrum

RESOURCE AVAILABILITY

Lead contact

Further information and requests for resources should be directed to and will be fulfilled by the lead contact, Dan Bang (danbang@cfin.au.dk).

Materials availability

This study did not generate new unique reagents.

Data and code availability

- De-identified neural, pupil, and behavioral data is available at GitHub (see key resources table).
- Code for reproducing figures is available at GitHub (see key resources table).
- Tutorial on HMM approach is available at GitHub (see key resources table).
- Other data and code are available upon request.

EXPERIMENTAL MODEL AND STUDY PARTICIPANT DETAILS

Patients

Six patients took part in the study; three patients performed the task (macro-micro; patient 1: female, 56 y; patient 2: female, 20 y; patient 3: male, 25 y) and three patients only contributed electrodes for *in vitro* evaluation (Behnke-Fried; patient 1: female, 62 y; patient 2: female, 27 y; patient 3: male, 28 y). The patients had medication-resistant epilepsy and undergone implantation of depth electrodes for localization of epileptic foci. Prior to the stay in the EMU, participation in the study was discussed with patients and the clinical team. The study protocol was described verbally and in a written format before patients provided verbal assent and written informed consent. No adverse or unanticipated events occurred during or as a result of the study protocol. The patient study was approved by the IRB committees at the Carilion Clinic (19-365) and Banner University Medical Center (STUDY00000295).

Controls

A cohort of seventeen adults (7 females, age range: 26-64 y) were recruited as controls (no reported history of psychiatric or neurological disorder) and performed the task in a standard laboratory setting. The control study was approved by the IRB committee at Virginia Tech (10-893).

METHOD DETAILS

Experimental task

Subjects performed a visual affective oddball task. The task was divided into six blocks of 100 images; each image was shown for 1 s and the images were separated by 1 s blank intervals. Within each block, 80% of the images were a checkerboard image (standard stimulus), whereas the remaining 20% were unique IAPS images (oddball stimuli). The presentation order was randomized within a block. The IAPS images were selected based on the image content ratings provided by the IAPS (Figure S2A) and grouped such that every block contained images of the same category: there were four emotionally evocative blocks, which varied in terms of valence (negative versus positive) and arousal (low versus high), and two emotionally neutral blocks. We included two neutral blocks to balance the design at the factor level (e.g., two neutral versus two high-arousal blocks). The order of the blocks was randomized within each subject. To keep subjects focused on the task, they were asked to press a button whenever an oddball stimulus was shown. The task was presented on a computer monitor mounted on top of the Tobii Pro Spectrum eye-tracking system. Subjects made a response by pressing any key on one of two button boxes, with a button box held in each hand.

Pupillometry

Data acquisition

The diameter of the pupil (pupil dilation) was monitored in the left and right eyes using the Tobii Pro Spectrum eye-tracking system running at a sampling rate of 1200 Hz. The subject was placed in a seated position 55-75 cm from the eye tracker as indicated by the Tobii Pro software. The system, which is fully external, was calibrated using a system-native 1 min protocol where the subject shifts their gaze to different corners of the computer screen.

Data pre-processing

The eye tracking system was unable to measure pupil diameter for some fraction of the samples taken, resulting in missing pupil diameter measurements. Sources of missing data include head movement, eyeblinks, and closing of the eyes. However, even when the eye tracking system is able to measure pupil diameter, the measurements may not always accurately reflect the actual pupil size, such as when the eyelid partly obscures the pupil. As is standard practice in pupillometry, we applied several pre-processing steps to reconstruct as much of the missing data as possible while correcting for as much of the invalid data as possible.

We adapted a velocity-based blink detection algorithm with cubic-spline reconstruction[45] for use with our specific eye-tracking system. In this algorithm, written in MATLAB, blinks are detected by the following sequence of features: (1) a high negative rate of change in pupil diameter (blink onset velocity); (2) a contiguous run of missing (or zero-valued) pupil diameter measurements (gap); (3) a high positive rate of change in pupil diameter (blink reversal velocity); and (4) a return to zero rate of change in pupil diameter (blink offset velocity). However, in our data, a large fraction of blinks did not exhibit the characteristic velocity excursions used by the original algorithm. To detect these blinks, we developed a second algorithm that detected gaps in pupil diameter measurements using the same gap duration thresholds as the velocity-based algorithm.

To reconstruct pupil diameter measurements across blinks, we also adapted the reconstruction itself. There was often significant noise in the pupil diameter measurements before and after blinks, likely induced by the eye-tracking system attempting to re-acquire the pupil. This issue required additional finesse in the reconstruction, resulting in these steps: (1) define a 5 s analysis window centered on the blink; (2) extract a smoothed (the "lowess" option to MATLAB's "smooth" function, 0.125 s window) pupil diameter vector from the analysis window; (3) calculate mean and standard deviation of the smoothed pupil diameter values outside of the blink; (4) find four equidistant points in the smoothed vector within 1/2 of the blink duration before the blink onset; (5) find four equidistant points in the smoothed vector within 1/2 of the blink duration after the blink offset; (6) calculate the cubic-spline interpolation of the eight points; (7) replace the (unsmoothed) blink with the interpolated values; (8) reject any reconstruction which would result in velocity excursions exceeding the thresholds used for blink detection; and (9) reject any reconstructions which would result in pupil diameters exceeding three standard deviations from the mean smoothed pupil diameter (from step 3).

Since the various thresholding parameters for the blink detection and reconstruction in the original algorithm were reported in terms of samples (at a sampling rate of 1 kHz) and arbitrary units of pupil size, we had to convert the values to milliseconds and millimeters respectively before we could apply the algorithm to our eye-tracking data. We found that using fixed velocity thresholds did not perform well, so we set our thresholds in terms of the standard deviation of pupil velocity after smoothing (see Table S1 for a summary of parameters and conversion from original algorithm). After blink detection and reconstruction, we still noticed small gaps in the pupil data. Any such gaps lasting 0.08 s or less were corrected by applying a simple shape-preserving cubic spline interpolation (the "pchip" option to MATLAB's "fillmissing" function). Before any data analysis, we averaged the reconstructed pupil data across the left and right eyes. If data was missing for one eye, then data from the other eye was used. Finally, the average pupil data was downsampled to 10 Hz using linear interpolation.

Data exclusion and quality

We excluded trials from pupil-related analyses when more than 50% of the data were reconstructed or missing following pre-processing (patient group: 24%; control group: 12%). For the included trials, 3% of the data were missing for a given time point in the patient group and 1% in the control group. While the proportion of excluded trials and missing time points is higher in the patient group, the presence of an oddball PDR in the patient group, and the correspondence between the oddball PDRs in the two groups, provide strong evidence for the reliability of the pupil data recorded in the EMU.

General description of electrochemical approach

Our approach builds on electrochemistry as applied in animals over the last three decades[46,47] and the recent adaptation of electrochemistry for use in the human brain.[15–18] However, the current study presents an advance in human electrochemistry, which to date has involved the insertion of research-exclusive carbon-fiber electrodes during awake DBS surgery, by showing that electrochemistry can be performed on depth electrodes that are already in standard clinical use. The data acquisition protocol in human electrochemistry is similar to fast-scan cyclic voltammetry (FSCV) as used in animals with regard to the time course of the voltage sweeps and the recording of the induced current time series during those sweeps.[48] The main change from animal work is the statistical method used to estimate the concentration of analytes of interest from the measured current time series.

FSCV involves the delivery of a rapid change in electrical potential to an electrode and measurement of the induced electrochemical reactions as changes in current at the electrode – with the guiding idea being that the current response carries information about both the identity and the concentration of analytes in the surrounding neural tissue. The goal of analysis of FSCV data is therefore to develop a statistical model that uses the current response in the best possible way to separate and estimate analytes of interest. The standard procedure is to train the statistical model on *in vitro* data collected in a laboratory setting where the presence and concentration of analytes of interest can be controlled and then apply this model to *in vivo* data for signal prediction.

Traditionally, the statistical model involves a decomposition of the *in vitro* training data into principal components that are then used for *in vivo* analyte inference within a regression framework.[49] In broad terms, this approach treats analyte inference as a problem of signal reconstruction: the concentration of an analyte of interest is estimated by mapping an *in vivo* current response onto those collected *in vitro* and then using the best match to label the *in vivo* current response. We instead treat analyte inference as a problem of signal prediction, with the statistical model optimized to generate accurate predictions about out-of-training data. Previous human work[16–18] has used elastic net regression,[50] but recent years have seen the development of more powerful machine learning methods. Here, we used deep convolutional neural networks as described below. Since information is distributed throughout a current time series and not only at the oxidation or reduction peaks typically revealed by principal components analysis,[16–18] we use non-decomposed data such that every time point within a current time series contributes to signal prediction. To facilitate out-of-training prediction, we train the model using large in vitro datasets and cross-validation as described below.

Earlier work has taken steps to validate this electrochemical approach. First, the human-compatible carbon-fiber electrodes have similar electrochemical properties to those used in the rodent brain.[15] Second, the signal prediction approach returns more reliable neuromodulator estimates than principal component regression.[16] Third, it does not confuse changes in pH for changes in neuromodulator levels.[16–18] Fourth, as shown here too (Figure S1), it can separate multiple neuromodulators from one another.[16–19] Fifth, it returns accurate neuromodulator estimates when tested in a laboratory setting where two neuromodulators change simultaneously across time.[18] However, since these validations have been performed *in vitro*, future work should explore *in vivo* validation in animals; for example, one could evaluate signal predictions under optogenetically-controlled neuromodulator release[38] and compare the time course of these predictions to that measured using fiber photometry.[39]

Implementation of electrochemistry in current study

Data acquisition

We conducted FSCV on standard Ad-Tech macro-micro electrodes (MM16A-SP05X-000) and Benhke-Fried electrodes (outer depth electrode: MM16A-SP05X-000; micro-wire bundle: WB09R-SP00X-0B6). The electrodes were stereotactically implanted into/explanted from the amygdala (macro-micro: left hemisphere in patient 1, right hemisphere in patients 2 and 3; Behnke-Fried: all left hemisphere). For the macro-micro electrodes, we used a longitudinally adjacent pair of micro-contacts on an electrode as our working and reference electrodes. For the Behnke-Fried electrodes, we used micro-wire #9 as our reference electrode and one of the other wires as our working electrode. Our FSCV protocol was a modification of previous work in both rodents[48,51] and humans.[15–19] Our measurement waveform was a standard triangular voltage ramp applied at 10 Hz

(macro-micro: hold at -0.6 V for 90 ms, ramp up from -0.6 V to +0.6 V at 240 V/s, ramp down from +0.6 V to -0.6 V at -240 V/s; Benhke-Fried: hold at -0.6 V for 90 ms, ramp up from -0.6 V to +0.175 V at 155 V/s, ramp down from +0.175 V to -0.6 V at -155 V/s) and we recorded the current response at 100 KHz. For *in vivo* experiments, we collected data for 2 min before the task to allow the electrode to equilibrate. We note that one of the macro-micro electrodes underwent changes in the current response during *in vitro* data collection. To maintain *in vitro* current traces similar to the *in vivo* ones, the triangular voltage ramp was decreased from ±240 V/s to ±160 V/s for all but the first *in vitro* dataset.

Signal prediction model

We generated *in vivo* signal predictions using an ensemble of deep convolutional neural networks that were trained and cross-validated on *in vitro* data with known concentrations of NA, DA, 5-HT and pH. The model architecture was based on the InceptionTime time series classification model[52] but modified for a regression framework. The model was implemented in Python,[40] using TensorFlow[43] and Keras.[44] Following previous applications,[52] equally weighted averages of *in vivo* signal predictions from multiple InceptionTime models were used to account for variability in the training process.

The (modified) InceptionTime model is based on two residual neural network (ResNet)[53] blocks, each containing three convolutional blocks. The input is added to the output of the first ResNet block, and, after activation, this serves as the input to the next ResNet block. It is also added to the output of the second ResNet block, and the sum flows through activation functions, global average pooling, and then finally to a dense layer with four output nodes, which after activation, gives the predictions of NA, DA, 5-HT, and pH. Each of the three convolutional layers in a ResNet block is further composed of convolutional blocks having four convolutional layers with 32 filters and increasing kernel sizes (1, 10, 20, and 40). The output of each of these convolutional layers is stacked together, after which batch normalization and activation are applied. This output serves as the input for the next convolutional block. All activation functions are RELU, except after the last dense layer, which uses softplus.

In vitro *training data*

We created specialized signal prediction models, by recovering the explanted electrode used for *in vivo* data collection and collecting *in vitro* data on this electrode for model training and evaluation. We collected four datasets. For each of the three neuromodulators (NA, DA, and 5-HT), a dataset was collected with 30 concentrations noisily dispersed over the range from 0 to 2500 nanomoles (nM) and with a pH of around 7.4. In addition, each of these datasets contained 5 mixture solutions in which the concentration of the neuromodulator in question was 840 or 1680 nM and one or two of the other neuromodulators had a concentration of 840 or 1680 nM. The fourth dataset was collected with 11 pH values in the range 7.0 to 7.8 and with the concentration of the three neuromodulators set to zero as well as 5 mixture solutions as described above. In each dataset, data collection was randomized across concentrations. For each concentration, the electrode was washed in PBS and then submerged in a PBS solution as described above. We applied our triangular voltage ramp at 10 Hz and recorded the current response at 100 KHz for 65 s. To reduce variation due to electrical noise and equilibration, we selected the most stable continuous 15 s section from the second half of the 65 s time window.

In vitro *model training*

For each electrode, a 10-fold cross-validation test was performed and then a signal prediction model was created. A training run involved a dataset being split into a training set containing around 90% of the data and a validation set containing the remainder. The data was split by concentration, such that all data points of any given concentration were in the same set. Before training, the training data were *Z* scored within analyte and then shifted by 10 standard deviations for each analyte to avoid zero gradients. After training, the predictions were then subjected to the inverse of the normalization procedure. A model was then trained on the training set using an ADAM optimizer,[54] with an initial learning rate of 1e-3, mean squared error loss and a batch size of 64. After each epoch, the loss on the validation set was calculated. If the performance on the validation set did not improve for five consecutive epochs, then the learning rate was halved until it reached a minimum of 1e-5. After 100 epochs were run, the model from the epoch with the lowest validation loss was selected as the final version. The training process was non-deterministic as variability is introduced by stochastic gradient descent, the initialization of the initial weights, and the order in which data is fed to the algorithm during training. When multiple training runs use the same data, different validation sets were used to reduce overfitting to any subset of the data.

In vitro *model evaluation*

To evaluate the sensitivity and specificity of the signal prediction models, a 10-fold cross-validation test was performed (Figures 1C and S1). The *in vitro* data was split into 10 discrete folds. Each fold was held out as a test set and an ensemble of four equally weighted sub-models was created via a training run on the remaining data. Ensembles of four models were used instead of five to reduce computation time. The mean of the predictions from each ensemble was calculated for its corresponding test set. All test sets were combined, resulting in all *in vitro* data points being predicted from ensembles that had never been trained or validated on data points with true concentrations of those being predicted.

In vivo *signal prediction*

In vivo signal predictions were generated using an ensemble of five equally weighted models from five training runs. No test set was held out to enable the model to train on the most data possible. *In vivo* current traces were submitted to each of the five models of the ensemble and the mean of the model predictions was used as the signal prediction.

QUANTIFICATION AND STATISTICAL ANALYSIS

Standardization

We standardized the data so that our group-level analyses could be performed in a relative frame of reference – where "large" and "small" values can be compared across patients. For example, for the analysis of the NA estimates shown in Figure 3A, we *Z* scored the trial-level NA estimates at the patient level, before running the linear mixed-effects regression at the group level. This approach minimizes, if not removes, the influence of unmodelled sources of patient-level variation in the baseline and/or the variance of the data.

Mixed-effects regression

All linear mixed-effects regression models consisted of fixed effects and a random intercept for each patient; model comparisons based on the Bayesian Information Criterion showed that the random-intercept models outperformed models which also included random effects. We applied the models at the trial level as this approach accommodates the difference in the number of standard and oddball trials. To illustrate our approach, we here consider the model for the estimated NA response around stimulus presentation. The model is specified as follows using Wilkinson notation:

$$NA \sim 1 + (arousal * valence + evocative) * type + (1 \mid subject)$$

In this model, stimulus type (standard = -1; oddball = 1) is specified at the trial level, and emotional valence (negative = -1; neutral = 0; positive = 1), emotional arousal (low = -1; neutral = 0; high = 1) and emotionally evocative (neutral = -1; evocative = 1) are specified at the block level as per our task design (Figure 2A). In addition, valence and arousal are considered features of blocks involving emotionally evocative IAPS images only (Figure S2A), with the evocative term capturing any difference between these blocks and blocks involving emotionally neutral IAPS images. We highlight that, because of our block design, a standard stimulus is not simply a non-IAPS image but a non-IAPS image of a particular category. This design feature is one of the reasons why it is possible to model interactions between stimulus type and the emotional variables.

Hidden Markov model

Hidden Markov models (HMMs), also known as Markov switching processes, are popular models for unsupervised statistical learning of multivariate time series data.[24,25] HMMs are especially suitable for data exhibiting non-stationary features that can be characterized by an underlying and unobserved hidden process. The approach assumes that the hidden process is in one of many latent states at any point in time, transitioning from one state to another over time, and that observations are generated from an emission distribution, conditional on the latent state sequence.

We implemented a bivariate HMM using R[41] and Stan.[42] The bivariate HMM was based on a discrete latent state sequence that partitions the pupil and NA estimates into different, potentially recurring regimes. Conditional on the latent state sequence, the two estimates are assumed to be generated from an emission distribution, here a bivariate Gaussian distribution with state-specific means and a state-specific variance-covariance matrix. The model allows a probabilistic estimate of the sequence of hidden states and the estimation of the state-specific parameters of the emission distribution.

Let $y_t = [NA(t), PD(t)]$ be the bivariate vector of the NA and pupil (PD) estimates at time point t, and let $z_t \in \{1, \ldots, K\}$ be the state of the hidden process at t, with K denoting the overall number of latent states. The generative mechanism of our proposed model is:

$$p(y_t | z_t = j) = N(\mu_j, \Sigma_j)$$

Where:

$$\mu_j = [mean(NA), mean(PD)]_j$$

$$\Sigma_j = \begin{bmatrix} var(NA) & cov(NA, PD) \\ cov(PD, NA) & var(PD) \end{bmatrix}.$$

We denote the state-specific transition probabilities as $\pi_j = [\pi_{j1}, \ldots, \pi_{jK}]$, with $\pi_{ji} = p(z_t = i | z_{t-1} = j)$, according to the Markovian property that the probability of a change at time t depends only upon the latent state at the previous time point $t - 1$.

We adopted a Bayesian approach to inference. Within the Bayesian framework, the model parameters are regarded as random variables and inference is carried out via their posterior distribution, which by Bayes' rule is proportional to the likelihood of the data times the prior distribution.[55] In our model, the relevant parameters are μ_j, Σ_j and π_j.

Our prior specification assumes a Dirichlet distribution on the transition probabilities π_j and Student's t distributions on the mean parameters μ_j. We further decompose the covariance matrix Σ_j into a vector of scale parameters τ_j and a correlation matrix Ω_j, and specify a half Student's t prior on τ_j, which has been truncated to admit only positive values, and an LKJ distribution on the Cholesky factor of Ω_j. Our prior specification is summarized as follows. For each state $j = 1, \ldots, J$

$$\pi_j \sim \text{Dirichlet}\,(1,1,1),$$
$$\mu_{ji} \sim \text{Student's}\; t\,(4;\;0,1.5),\; i \in \{\text{NA},\text{PD}\}$$
$$\tau_{ji} \sim \text{Student's}\; t^{+}(4;\;1,1.5),$$
$$L_{\Omega_j} \sim \text{LKJ}(1)$$

where π_j is the vector of transition probabilities, and $\mu_j = (\mu_{jNA}, \mu_{jPD})$ and $\tau_j = (\tau_{jNA}, \tau_{jPD})$ represent the vectors of state specific means and scales. The superscript $^{+}$ indicates that the distribution has been truncated to admit only positive values, and LKJ indicates the LKJ prior,[56] a family of probability distribution for positive definite correlation matrices (or equivalently for their Cholesky factors).

The posterior distribution is not available in closed form; we therefore used the No-U-Turn Sampler (NUTS) which leverages Hamiltonian dynamics to draw samples from the joint posterior distribution of the model parameters.

The number of latent states, K, for an HMM was selected by inspecting the posterior predictive fits and, in a more principled way, by calculating the ratio of marginal likelihoods from different models. The latter is often called the Bayes factor,[57] and can be thought of as the weight of evidence in favor of a model against a competing one. Bridge sampling[58] provides a general procedure for estimating marginal likelihoods in a reliable manner. This estimator can be implemented in R using the package *bridgesampling*,[59] whose compatibility with Stan makes it particularly straightforward to estimate the marginal likelihood directly from a Stan output.

Current Biology, Volume *33*

Supplemental Information

Noradrenaline tracks emotional modulation

# of attention in human amygdala

Dan Bang, Yi Luo, Leonardo S. Barbosa, Seth R. Batten, Beniamino Hadj-Amar, Thomas Twomey, Natalie Melville, Jason P. White, Alexis Torres, Xavier Celaya, Priya Ramaiah, Samuel M. McClure, Gene A. Brewer, Robert W. Bina, Terry Lohrenz, Brooks Casas, Pearl H. Chiu, Marina Vannucci, Kenneth T. Kishida, Mark R. Witcher, and P. Read Montague

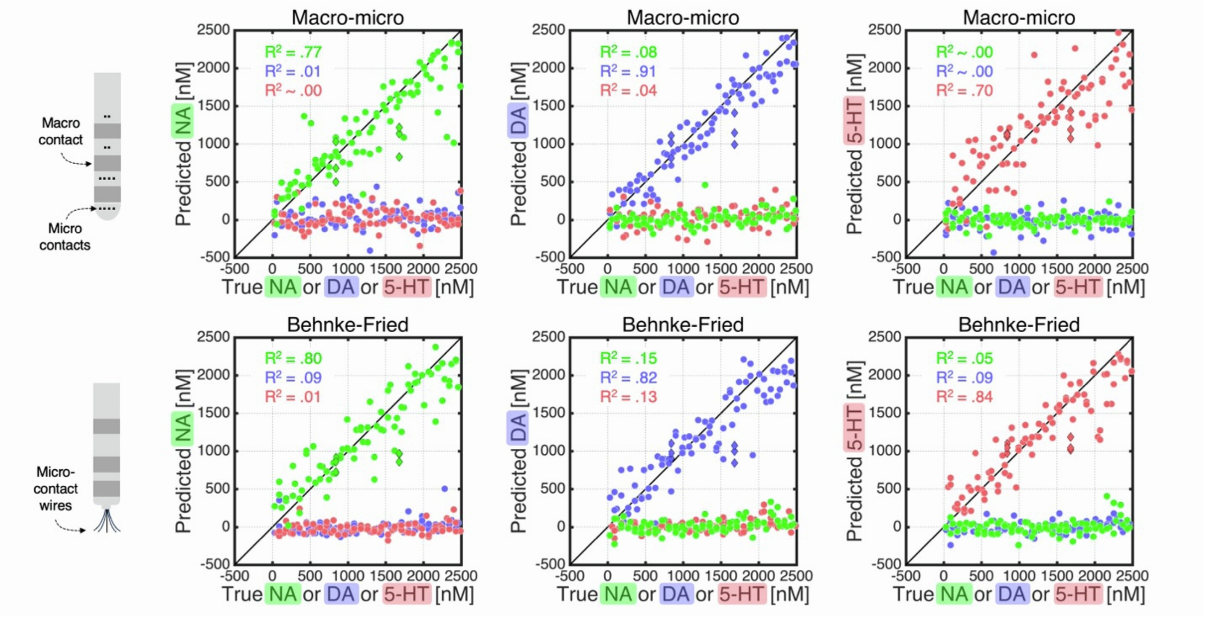

Figure S1. ***In vitro*** **evaluation of electrochemical approach for NA, DA, and 5-HT. Related to Figure 1.**

The macro-micro electrodes were explanted from the three patients who performed the task in Figure 2A. The Behnke-Fried electrodes were explanted from the amygdala of three patients at another hospital and were included to demonstrate generalizability. Dots indicate the average predicted (left) NA, (middle) DA, or (right) 5-HT concentration (nanomoles, nM) for single-analyte solutions which only contained NA (green), DA (blue), or 5-HT (red). Diamonds indicate mixture solutions which contained other neuromodulators in addition to the one being predicted. Predictions were from a 10-fold cross-validation and pooled across patients. R^2-values were obtained by regressing the predicted concentration against the true NA, DA, or 5-HT concentration. Error bars represent 95% confidence intervals but are not visible at this scale.

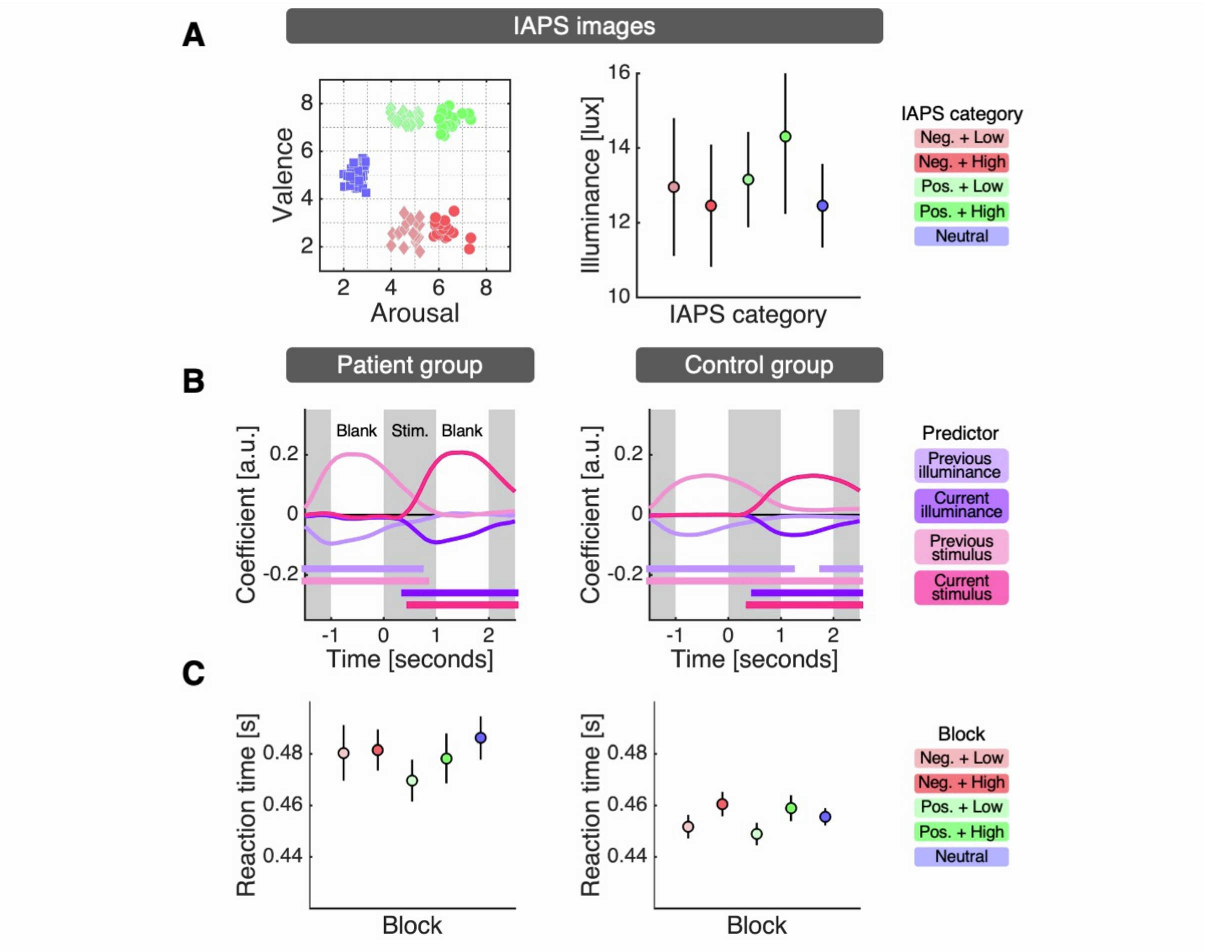

Figure S2. IAPS images, pupil dilation, and response times. Related to Figure 2.

(A) The sub-panels show (left) the arousal and valence ratings provided by the IAPS and (right) the illuminance for the different image sets. The mean ± SD valence (V) and arousal (A) ratings were: "Neg. + Low", $V = 2.6 \pm 0.4$, $A = 4.8 \pm 0.4$; "Neg. + High", $V = 2.7 \pm 0.4$, $A = 6.3 \pm 0.4$; "Pos. + Low", $V = 7.4 \pm 0.2$, $A = 4.6 \pm 0.4$; "Pos. + High", $V = 7.2 \pm 0.4$, $A = 6.5 \pm 0.4$; "Neutral", $V = 5.0 \pm 0.4$, $A = 2.6 \pm 0.2$. The neutral images do not neatly map onto the two-dimensional valence x arousal space: the two dimensions collapse into a single dimension in the sense that one cannot have low- or high-arousal images of neutral valence. The images can therefore be thought of as being drawn from two groups – emotionally evocative versus emotionally neutral – with valence and arousal being a property of emotionally evocative images. Illuminance (in units of lux) measures the total amount of light that falls on the eye and did not differ between image sets (independent-samples t-test, all absolute $t < 0.86$, all $p > 0.380$. The illuminance of the checkerboard image used as the standard stimulus was higher than the IAPS images (lux: 18). The illuminance data are represented as mean ± SE across images.

(B) Coefficients from time point-by-time point linear mixed-effects regressions for (left) patient group and (right) control group where we predicted temporally smoothed but non-standardized pupil estimates using stimulus type and illuminance for the current and previous trial. Note (1) that the effects of the previous trials linger into the current trial and (2) that the effects of the current trial arise after the presentation of the current stimulus. Squares indicate the significance ($p < 0.050$) of the predictor associated with the shown color.

(C) Reaction times for (left) patient group and (right) control group separated by block. We ran a linear mixed-effects regression for oddball trials in which we predicted trial-by-trial reaction times (Z score after log-transform) using valence, arousal, evocative and the interaction between valence and arousal. This analysis did not return any effects for the patient group (all absolute $t < 0.79$, all $p > 0.430$). However, in line with the NA results, it identified an effect of arousal for the control group, with participants being slower to respond to oddball stimuli in the high-arousal blocks (arousal, $t(2025) = 2.25$, $p = 0.025$; all other absolute $t < 0.86$, all other $p > 0.390$). One reason for the subtlety of the reaction time effects is likely to be the regular inter-trial interval which may have made responses easy to prepare and thereby may have reduced the opportunity for cognitive factors to affect reaction times. Data are represented as mean ± SE across trials.

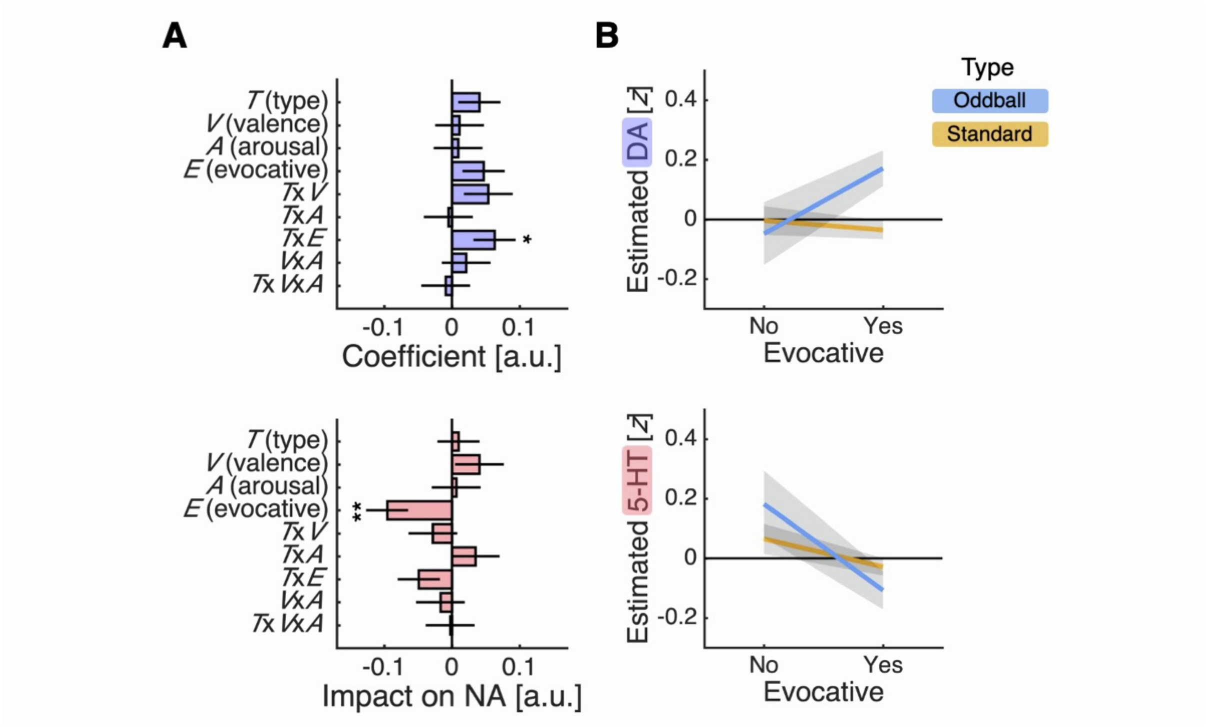

Figure S3. DA and 5-HT track emotionally evocative versus emotionally neutral blocks. Related to Figure 3.

(A) Regression coefficients ± SE from a linear mixed-effects regression in which we predicted single-trial (top) DA or (bottom) 5-HT estimates using stimulus type, emotional valence, emotional arousal*,* emotionally evocative, and interactions between these terms. For DA, we found a positive interaction between stimulus type and emotionally evocative (type x evocative; $t(1790) = 2.01$, $p = 0.044$). Simple-effects analysis showed that the estimated DA response for oddball stimuli was higher in emotionally evocative than emotionally neutral blocks (panel B; effect of evocative for oddball stimuli, $t(1790) = 1.97$, $p \sim 0.049$), whereas the estimated DA response for standard stimuli did not differ between these blocks (panel B; effect of evocative for standard stimuli, $t(1790) = -0.57$, $p = 0.568$). Indicating that these effects also reflect contextual modulation of the attentional salience of surprising stimuli, we found that the estimated DA response was higher for oddball than standard stimuli in emotionally evocative blocks but similar in emotionally neutral blocks (Figure 3B; effect of type for evocative, $t(1790) = 2.87$, $p = 0.004$; effect of type for neutral, $t(1790) = -0.44$, $p = 0.663$). For 5-HT, we found a negative main effect of emotionally evocative (evocative, $t(1790) = -3.06$, $p = 0.002$), indicating that the estimated 5-HT response was overall higher in emotionally neutral blocks. *: $p < 0.050$; **: $p < 0.010$; ***: $p < 0.001$.

(B) Single-trial (top) DA and (bottom) 5-HT estimates (mean ± SE) separated by whether a block is emotionally evocative and stimulus type.

(A and B) Single-trial NA estimates were calculated as the mean NA estimate over a 1 s window centered on stimulus onset minus the mean NA estimate over the preceding 0.5 s and *Z* scored across trials for each patient.

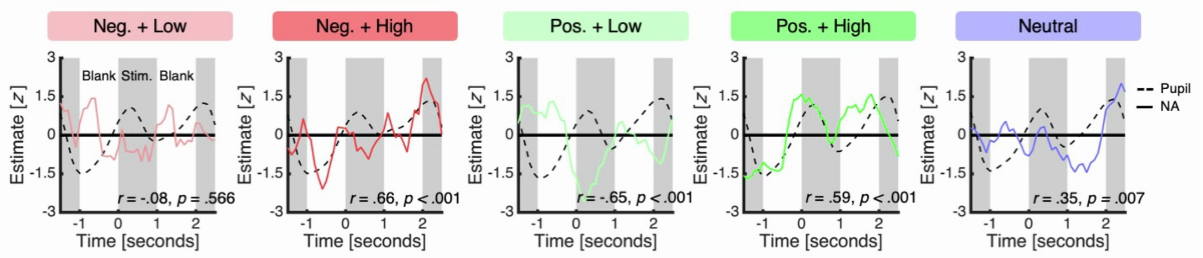

Figure S4. Simple correlation between pupil and NA estimates for oddball stimuli in each block. Related to Figure 4.

The panels show the average estimated pupil and NA time series with simple correlation statistics reported for oddball stimuli in each block. We first smoothed the time series using a 0.5 s causal filter and *Z* scored the smoothed data for each trial. We then averaged the time series for each condition, removed any linear drift and scaled the average time series to have unit variance, by dividing each time point by the average of the condition-specific standard deviations over the average time series. The last step helps ensure that estimated correlations, which are sensitive to the variance of the data, are comparable across conditions.

Parameter	Original	Converted	Used
Blink onset velocity threshold	10 a.u. per sample	0.0124 mm per ms	$3 \cdot \sigma_v$
Blink reversal velocity threshold	5 a.u. per sample	0.0062 mm per ms	$1.5 \cdot \sigma_v$
Minimum gap duration	100 samples	100 ms	100 ms
Maximum gap duration	500 samples	500 ms	600 ms
Blink reconstruction margin	10 samples	10 ms	20 ms
Smoothing window size	21 samples	21 ms	20.833 ms

Table S1. Pupil pre-processing parameters. Related to STAR Methods.

Summary of parameters in pupil pre-processing pipeline. The original values refer to the values used in the original implementation of the velocity-based blink detection algorithm with cubic-spline reconstruction.
