# Supplementary material for: Noradrenaline tracks emotional modulation of attention in human amygdala
Source: Curr Biol. 2023 Nov 20;33(22):5003–5010.e6. doi: 10.1016/j.cub.2023.09.074 (PMC10957395; doi:10.1016/j.cub.2023.09.074)
Supplement: Supplementary file 1 [file mmc1.pdf]

Current Biology, Volume *33*

Supplemental Information

Noradrenaline tracks emotional modulation of attention in human amygdala

Dan Bang, Yi Luo, Leonardo S. Barbosa, Seth R. Batten, Beniamino Hadj-Amar, Thomas Twomey, Natalie Melville, Jason P. White, Alexis Torres, Xavier Celaya, Priya Ramaiah, Samuel M. McClure, Gene A. Brewer, Robert W. Bina, Terry Lohrenz, Brooks Casas, Pearl H. Chiu, Marina Vannucci, Kenneth T. Kishida, Mark R. Witcher, and P. Read Montague

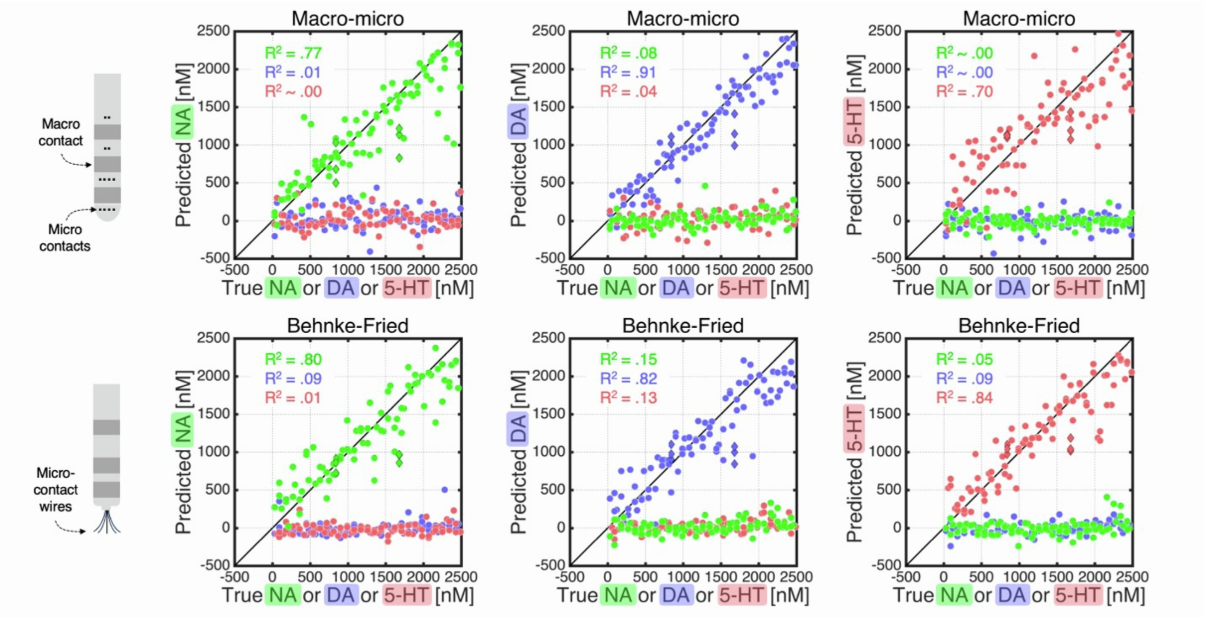

Figure S1. ***In vitro*** **evaluation of electrochemical approach for NA, DA, and 5-HT. Related to Figure 1.**

The macro-micro electrodes were explanted from the three patients who performed the task in Figure 2A. The Behnke-Fried electrodes were explanted from the amygdala of three patients at another hospital and were included to demonstrate generalizability. Dots indicate the average predicted (left) NA, (middle) DA, or (right) 5-HT concentration (nanomoles, nM) for single-analyte solutions which only contained NA (green), DA (blue), or 5-HT (red). Diamonds indicate mixture solutions which contained other neuromodulators in addition to the one being predicted. Predictions were from a 10-fold cross-validation and pooled across patients. R^2-values were obtained by regressing the predicted concentration against the true NA, DA, or 5-HT concentration. Error bars represent 95% confidence intervals but are not visible at this scale.

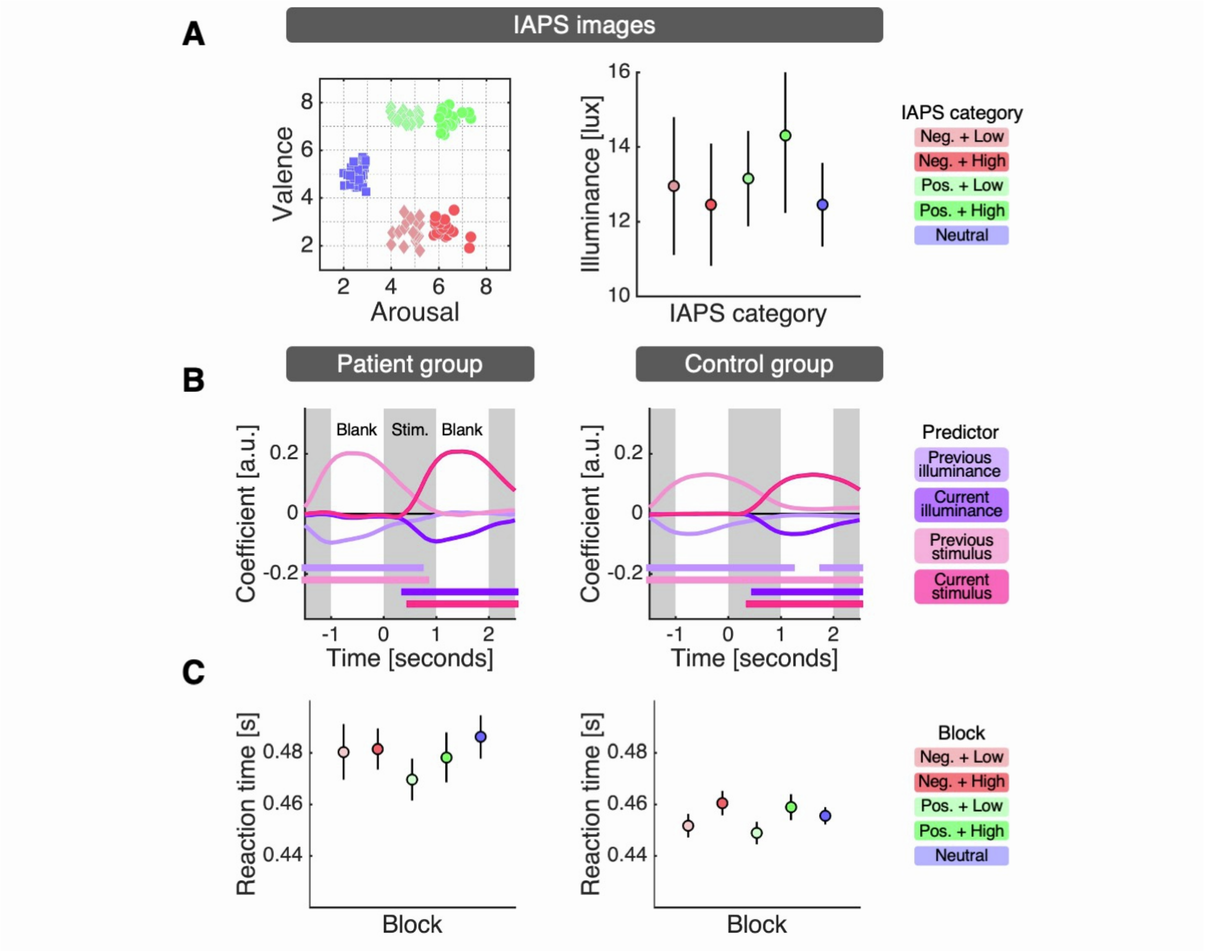

Figure S2. IAPS images, pupil dilation, and response times. Related to Figure 2.

(A) The sub-panels show (left) the arousal and valence ratings provided by the IAPS and (right) the illuminance for the different image sets. The mean ± SD valence (*V*) and arousal (*A*) ratings were: "Neg. + Low", *V* = 2.6 ± 0.4, *A* = 4.8 ± 0.4; "Neg. + High", *V* = 2.7 ± 0.4, *A* = 6.3 ± 0.4; "Pos. + Low", *V* = 7.4 ± 0.2, *A* = 4.6 ± 0.4; "Pos. + High", *V* = 7.2 ± 0.4, *A* = 6.5 ± 0.4; "Neutral", *V* = 5.0 ± 0.4, *A* = 2.6 ± 0.2. The neutral images do not neatly map onto the two-dimensional valence x arousal space: the two dimensions collapse into a single dimension in the sense that one cannot have low- or high-arousal images of neutral valence. The images can therefore be thought of as being drawn from two groups – emotionally evocative versus emotionally neutral – with valence and arousal being a property of emotionally evocative images. Illuminance (in units of lux) measures the total amount of light that falls on the eye and did not differ between image sets (independent-samples *t*-test, all absolute $t < 0.86$, all $p > 0.380$. The illuminance of the checkerboard image used as the standard stimulus was higher than the IAPS images (lux: 18). The illuminance data are represented as mean ± SE across images.

(B) Coefficients from time point-by-time point linear mixed-effects regressions for (left) patient group and (right) control group where we predicted temporally smoothed but non-standardized pupil estimates using stimulus type and illuminance for the current and previous trial. Note (1) that the effects of the previous trials linger into the current trial and (2) that the effects of the current trial arise after the presentation of the current stimulus. Squares indicate the significance ($p < 0.050$) of the predictor associated with the shown color.

(C) Reaction times for (left) patient group and (right) control group separated by block. We ran a linear mixed-effects regression for oddball trials in which we predicted trial-by-trial reaction times (*Z* score after log-transform) using valence, arousal, evocative and the interaction between valence and arousal. This analysis did not return any effects for the patient group (all absolute $t < 0.79$, all $p > 0.430$). However, in line with the NA results, it identified an effect of arousal for the control group, with participants being slower to respond to oddball stimuli in the high-arousal blocks (arousal, $t(2025) = 2.25$, $p = 0.025$; all other absolute $t < 0.86$, all other $p > 0.390$). One reason for the subtlety of the reaction time effects is likely to be the regular inter-trial interval which may have made responses easy to prepare and thereby may have reduced the opportunity for cognitive factors to affect reaction times. Data are represented as mean ± SE across trials.

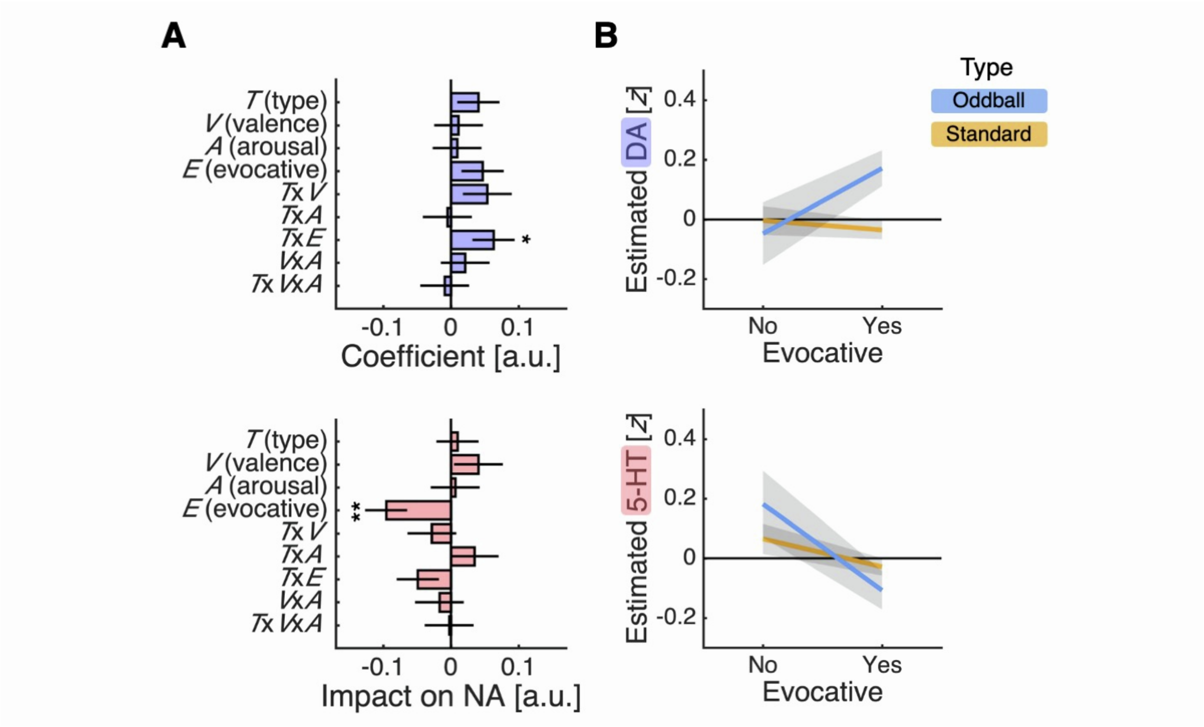

Figure S3. DA and 5-HT track emotionally evocative versus emotionally neutral blocks. Related to Figure 3.

(A) Regression coefficients ± SE from a linear mixed-effects regression in which we predicted single-trial (top) DA or (bottom) 5-HT estimates using stimulus type, emotional valence, emotional arousal, emotionally evocative, and interactions between these terms. For DA, we found a positive interaction between stimulus type and emotionally evocative (type x evocative; $t(1790) = 2.01$, $p = 0.044$). Simple-effects analysis showed that the estimated DA response for oddball stimuli was higher in emotionally evocative than emotionally neutral blocks (panel B; effect of evocative for oddball stimuli, $t(1790) = 1.97$, $p \sim 0.049$), whereas the estimated DA response for standard stimuli did not differ between these blocks (panel B; effect of evocative for standard stimuli, $t(1790) = -0.57$, $p = 0.568$). Indicating that these effects also reflect contextual modulation of the attentional salience of surprising stimuli, we found that the estimated DA response was higher for oddball than standard stimuli in emotionally evocative blocks but similar in emotionally neutral blocks (Figure 3B; effect of type for evocative, $t(1790) = 2.87$, $p = 0.004$; effect of type for neutral, $t(1790) = -0.44$, $p = 0.663$). For 5-HT, we found a negative main effect of emotionally evocative (evocative, $t(1790) = -3.06$, $p = 0.002$), indicating that the estimated 5-HT response was overall higher in emotionally neutral blocks. *: $p < 0.050$; **: $p < 0.010$; ***: $p < 0.001$.

(B) Single-trial (top) DA and (bottom) 5-HT estimates (mean ± SE) separated by whether a block is emotionally evocative and stimulus type.

(A and B) Single-trial NA estimates were calculated as the mean NA estimate over a 1 s window centered on stimulus onset minus the mean NA estimate over the preceding 0.5 s and Z scored across trials for each patient.

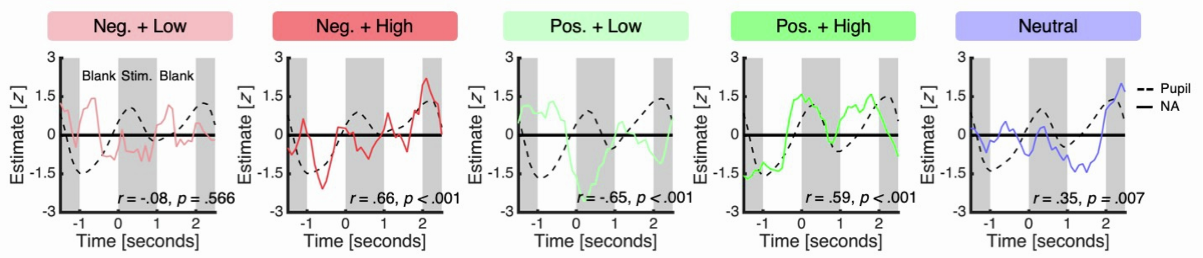

Figure S4. Simple correlation between pupil and NA estimates for oddball stimuli in each block. Related to Figure 4.

The panels show the average estimated pupil and NA time series with simple correlation statistics reported for oddball stimuli in each block. We first smoothed the time series using a 0.5 s causal filter and *Z* scored the smoothed data for each trial. We then averaged the time series for each condition, removed any linear drift and scaled the average time series to have unit variance, by dividing each time point by the average of the condition-specific standard deviations over the average time series. The last step helps ensure that estimated correlations, which are sensitive to the variance of the data, are comparable across conditions.

Parameter	Original	Converted	Used
Blink onset velocity threshold	10 a.u. per sample	0.0124 mm per ms	$3 \cdot \sigma_v$
Blink reversal velocity threshold	5 a.u. per sample	0.0062 mm per ms	$1.5 \cdot \sigma_v$
Minimum gap duration	100 samples	100 ms	100 ms
Maximum gap duration	500 samples	500 ms	600 ms
Blink reconstruction margin	10 samples	10 ms	20 ms
Smoothing window size	21 samples	21 ms	20.833 ms

Table S1. Pupil pre-processing parameters. Related to STAR Methods.

Summary of parameters in pupil pre-processing pipeline. The original values refer to the values used in the original implementation of the velocity-based blink detection algorithm with cubic-spline reconstruction.
